# Exosome-mediated miR-146a transfer suppresses type I interferon response and facilitates EV71 infection

**DOI:** 10.1371/journal.ppat.1006611

**Published:** 2017-09-14

**Authors:** Yuxuan Fu, Li Zhang, Fang Zhang, Ting Tang, Qi Zhou, Chunhong Feng, Yu Jin, Zhiwei Wu

**Affiliations:** 1 Center for Public Health Research, Medical School, Nanjing University, Nanjing, PR China; 2 Nanjing Children's Hospital, Nanjing Medical University, Nanjing, PR China; 3 State Key Lab of Analytical Chemistry for Life Science, Nanjing University, Nanjing, PR China; 4 Medical School and Jiangsu Key Laboratory of Molecular Medicine, Nanjing University, Nanjing, PR China; University of Pittsburgh, UNITED STATES

## Abstract

Exosomes can transfer genetic materials between cells. Their roles in viral infections are beginning to be appreciated. Researches have shown that exosomes released from virus-infected cells contain a variety of viral and host cellular factors that are able to modulate recipient’s cellular response and result in productive infection of the recipient host. Here, we showed that EV71 infection resulted in upregulated exosome secretion and differential packaging of the viral genomic RNA and miR-146a into exosomes. We provided evidence showing that miR-146a was preferentially enriched in exosomes while the viral RNA was not in infected cells. Moreover, the exosomes contained replication-competent EV71 RNA in complex with miR-146a, Ago2, and GW182 and could mediate EV71 transmission independent of virus-specific receptor. The exosomal viral RNA could be transferred to and replicate in a new target cell while the exosomal miR-146a suppressed type I interferon response in the target cell, thus facilitating the viral replication. Additionally, we found that the IFN-stimulated gene factors (ISGs), BST-2/tetherin, were involved in regulating EV71-induced upregulation of exosome secretion. Importantly, *in vivo* study showed that exosomal viral RNA exhibited differential tissue accumulation as compared to the free virus particles. Together, our findings provide evidence that exosomes secreted by EV71-infected cells selectively packaged high level miR-146a that can be functionally transferred to and facilitate exosomal EV71 RNA to replicate in the recipient cells by suppressing type I interferon response.

## Author summary

Exosomes are small membrane-encapsulated vesicles that secrete into the extracellular environment. Various proteins and RNA molecules have been identified in exosomes whose content reflects the physiological or pathological state of the host cells. Researches have shown that exosomes released from virus-infected cells contain a variety of viral and host cellular factors that are able to modulate recipient’s cellular responses and result in productive infection of the recipient host. Here, we showed that Enterovirus 71 (EV71), a non-enveloped, single-strand positive sense RNA virus that belongs to the family *Picornaviridae* and is a major etiologic agent of hand-foot and-mouth disease (HFMD), could stimulate exosome secretion and differential packaging of the viral genomic RNA and miR-146a into exosomes. The exosomal viral RNA could be transferred to and replicate in a new target cell while the exosomal miR-146a suppressed type I interferon response in the target cell, thus facilitating the viral replication. Importantly, *in vivo* study showed that exosomal viral RNA exhibited differential tissue accumulation as compared to the free virus particles. We postulate that the preferential packaging of miRNA-146a into exosome is a viral strategy of suppressing host innate immunity upon infection and the exosomal EV 71 RNA may play an important pathogenic role in the infection.

## Introduction

Exosomes are small membrane-encapsulated vesicles that secrete into the extracellular environment. Various proteins and RNA molecules have been identified in exosomes whose content reflects the physiological or pathological state of the host cells. The exosome can deliver its content, such as proteins, lipids, and RNAs, to distal tissues or cells and participate in various biological processes [1–3]. One of the main exosomal functions is to mediate intercellular communication during viral pathogenesis and immune responses [4, 5]. Exosomes have been shown to excrete from cells infected by viruses and deliver viral particles, genomes, and other host genetic elements to neighboring cells, helping to establish productive infections and modulating host immune response [6–9]. For instance, exosomes released from HCV-infected cells contain full-length viral RNA, which can be successfully transferred to dendritic cells to establish productive infection [7, 10]; Human T-lymphotropic virus type 1 (HTLV-1)-infected cells contain Tax, HBZ, and Env mRNA transcripts, suggesting that exosomes can serve as vehicles to deliver functional HTLV-1 mRNA to recipient cells [8]; Recently, it has been shown that exosomes released from HIV-infected cells contain negative regulatory factor, which induces apoptosis of uninfected cells [11]. Meanwhile, exosomes can protect their contents from immune recognition, which appears especially important for non-enveloped viruses [12]. These observations suggest crucial roles for exosomes in the viral life cycle; however, how exosomes regulate host immunity and impact on viral infection is less established.

Exosomal RNAs have been implicated in many exosome-mediated biological functions. RNA sequencing analysis has shown that there is a diverse collection of the exosomal RNA species, among which microRNAs (miRNAs) are the most abundant [13]. Recent evidence showed that multiple cell types, such as immune cells, endothelial cells (ECs) and cancer cells can both secrete and take up exosomal miRNAs [14–16]. Modification of the exosome composition of host-derived miRNAs by viral products can significantly affect the biological activities mediated by taken-up exosomes [14, 15]. Importantly, studies demonstrated that the loading of miRNAs into exosomes was a selective process and did not simply reflect the dysregulated miRNA composition in parental cells [17–19]. It is conceivable that the exosome-mediated intercellular transfer of miRNAs allows viruses to modulate immune defense of the neighboring cells and facilitates the spread of virus [4, 5].

Enterovirus 71 (EV71), a non-enveloped, single-strand positive sense RNA virus that belongs to the family *Picornaviridae*, is a major etiologic agent of hand-foot and-mouth disease (HFMD) [20, 21]. Infection is sometimes associated with extensive neuronal degeneration, severe CNS inflammation and necrosis, and pulmonary congestion and hemorrhaging. However, autopsies suggested that no significant inflammation was found in organs, such as the lungs, heart, or pancreas [22–27]. The mechanism of EV71 pathogenesis remains unclear. It is believed that host factors especially host immune response rather than EV71 itself or its genotype may be one of the important determinants for the disease severity [28]. Excessive proinflammatory cytokine and chemokine responses were considered to contribute to the severity of EV71 infection [29]. EV71 infection does not induce production of type I interferon (IFN) [30]. Recently, EV71 viral RNA has been identified in the exosomes derived from EV71-infected rhabdomyosarcoma cells, resulting in productive infection of a human neuroblastoma cell line [31]. However, the molecular mechanisms that control exosome composition and content during EV71 infection are still not fully understood.

In the current study, we showed that EV71 infection upregulated exosome secretion and resulted in differential packaging of the viral genomic RNA and miR-146a, a cellular miRNA that is known to suppress type I interferon response and impact on EV71 infection. We provided evidence showing that miR-146a was preferentially co-packaged with EV71 genomic RNA in exosomes. The exosomal viral RNA could be transferred to and replicate in a recipient cell while the exosomal miR-146a could suppress type I interferon expression in the target cell, thus facilitating the viral replication. Moreover, we found that the IFN-stimulated gene factors (ISGs), BST-2/tetherin, were involved in regulating EV71-induced upregulation of exosome secretion. Importantly, *in vivo* study showed that exosome-containing viral RNA exhibited differential tissue accumulation distinct from free virus particles.

## Results

### EV71 infection increased exosome secretion

**Fig 1:**
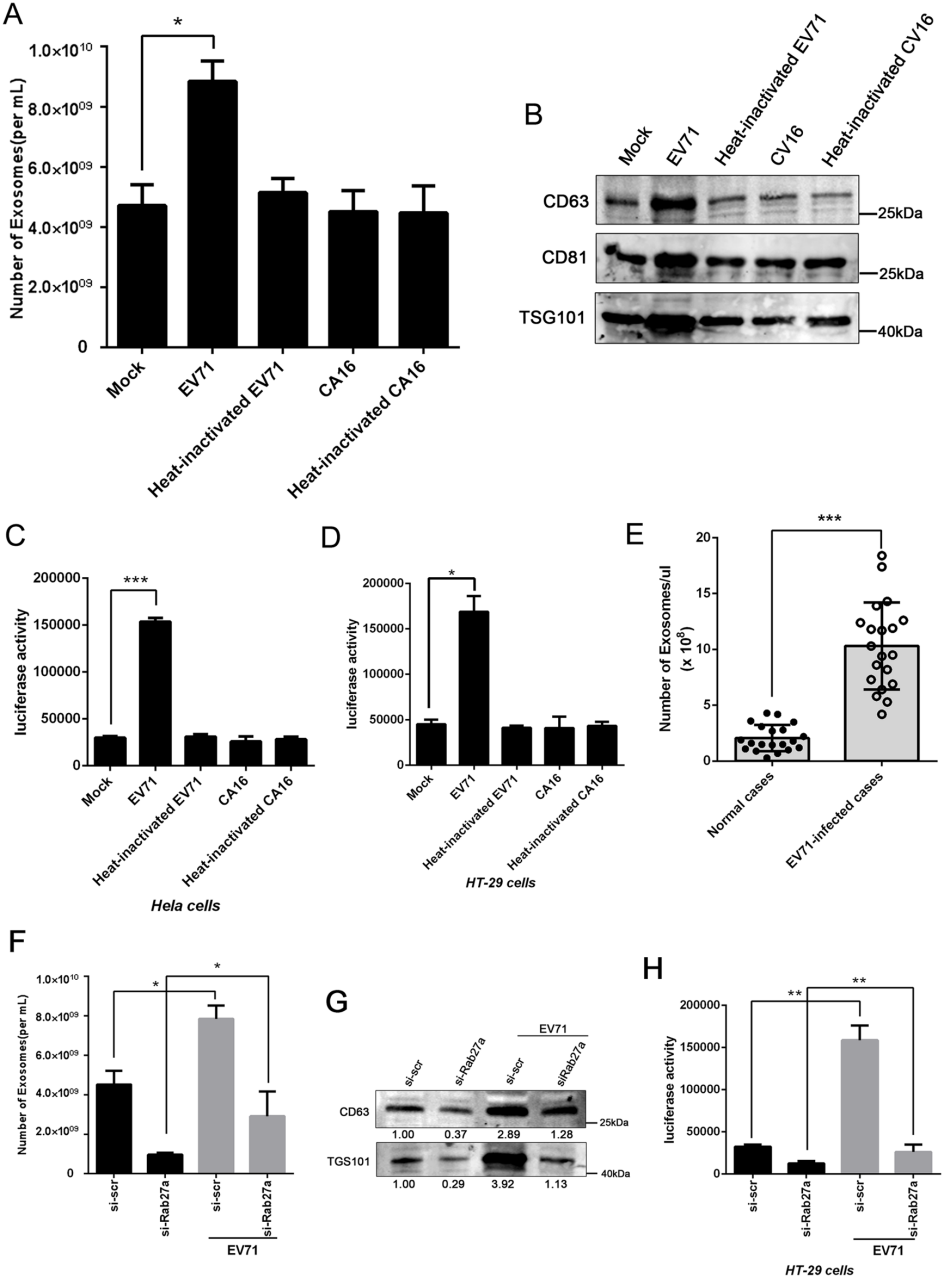
EV71 infection increased exosome secretion. (A and B)Exosomes were isolated by Exoquick+CD63 immuno-magnetic selection. Quantification of exosomes purified from THP-1 or HT-29 cells with various treatments was carried out by Nanoparticle Tracking Analysis (NTA) (A) and Western blot (B). Data are shown as mean±SEM of three independent purifications. (C and D) Exosome secretion induced by EV71 or CA16 infection was monitored by luciferase activity of CD63. Data are shown as mean±SEM of three independent experiments. (E) Total immuno-magnetic CD63 selected exosomes were isolated from sera of EV71 infected (n = 20) or uninfected (n = 20) individuals and quantified using NTA. (F and G): Exosomes purified from THP-1 or HT-29 cells after be transfected with control siRNA (si-scr) or Rab27a siRNA (si-Rab27a) for 24h, followed by infection with EV71 at 0.05 or 0.1 TCID_50_ respectively. The quantification of exosomes with various treatments was carried out by Nanoparticle Tracking Analysis (NTA) (F) and Western blot (G). Data are shown as mean±SEM of three independent experiments. The numbers represent the relative density of the bands relative to the corresponding si-scr group. Value of si-scr group is set at 1.00 (100%). (H): Exosome secretion induced by EV71 was monitored by luciferase activity of CD63. Data are shown as mean±SEM of three independent purifications. (*p<0.05, ***p<0.001).

We observed that EV71 infection upregulated exosome production. We isolated exosomes from the supernatants of the mock, EV71, heat-inactivated EV71 and coxsackie virus A16 (CA16) infected cells and determined the relative quantity of exosomes secreted. To ensure purity and homogeneity of the exosomes, we optimized a two-step purification approach by using Exoquick-TC combining with CD63 immuno-magnetic isolation. The free virus (Free-EV71) was present in the supernatant following Exoquick centrifugation and flow through following CD63 immuno-magnetic isolation (S1A Fig). The morphology of the isolated exosomes was verified by electron microscopy and Nanoparticle Tracking Analysis (NTA) (S1B and S1C Fig). It was clear that both EV71-infected THP-1 and HT-29 cells had a significant increase in the exosomes secreted as determined by NTA and Western blot (Fig 1A and 1B). We calculated that one uninfected THP-1 or HT-29 cell secreted ~2500 or ~800 exosomes within 24h of culture, respectively; however, EV71-infected THP-1 and HT-29 cells increased the exosome secretion by 2.4- and 2.2-fold, respectively (S1D and S1E Fig). In contrast, the cells infected with heat-inactivated EV71, CA16 or hear-inactivated CA16 showed no difference in exosome secretion from the mock-infected cells.

To further quantify the exosome secretion, we generated stable, inducible Hela and HT-29 cell lines expressing a CD63-luciferase fusion protein. The CD63-luciferase cell line was then used to monitor exosome secretion upon EV71 or CA16 infection. As shown in Fig 1C and 1D, luciferase activity increased by 5-fold and 3.5-fold in Hela and HT-29 cells infected with EV71, respectively, while no significant changes were observed in cells infected with heat-inactivated EV71, CA16 or heat-inactivated CA16 as compared to the mock infection. These observations also suggest that EV71 infection but not CA16 could enhance exosome production. This observation was also supported by clinical data as we quantified the serum exosome in a cohort of EV71-infected patients (number: 20) and uninfected children (number: 20). Significantly higher levels of exosomes were found in the sera of infected children than the uninfected, as shown in Fig 1E. To confirm that it is actually an increase of exosomes specifically and not other extracellular vesicles, THP-1 and HT-29 cells were treated before EV71 infection with small interfering RNA (siRNA) against Rab27a (si-Rab27a), a Rab GTPase required for the exosome secretion [32]. Our results showed that both EV71-infected THP-1 and HT-29 cells had a significant decrease in the exosomes secreted before being treated with si-Rab27a as compared to that of the cells transfected with scrambled control siRNA (si-scr) determined by NTA (Fig 1F) and Western blot (Fig 1G). Similar result was confirmed by luciferase activity assay on CD63-luciferase HT-29 cell line (Fig 1H). Taken together, our findings confirmed that EV71 infection could induce exosome secretion from host cells.

### Characterization of exosomal EV71 infectivity *in vitro* and *in vivo*

**Fig 2:**
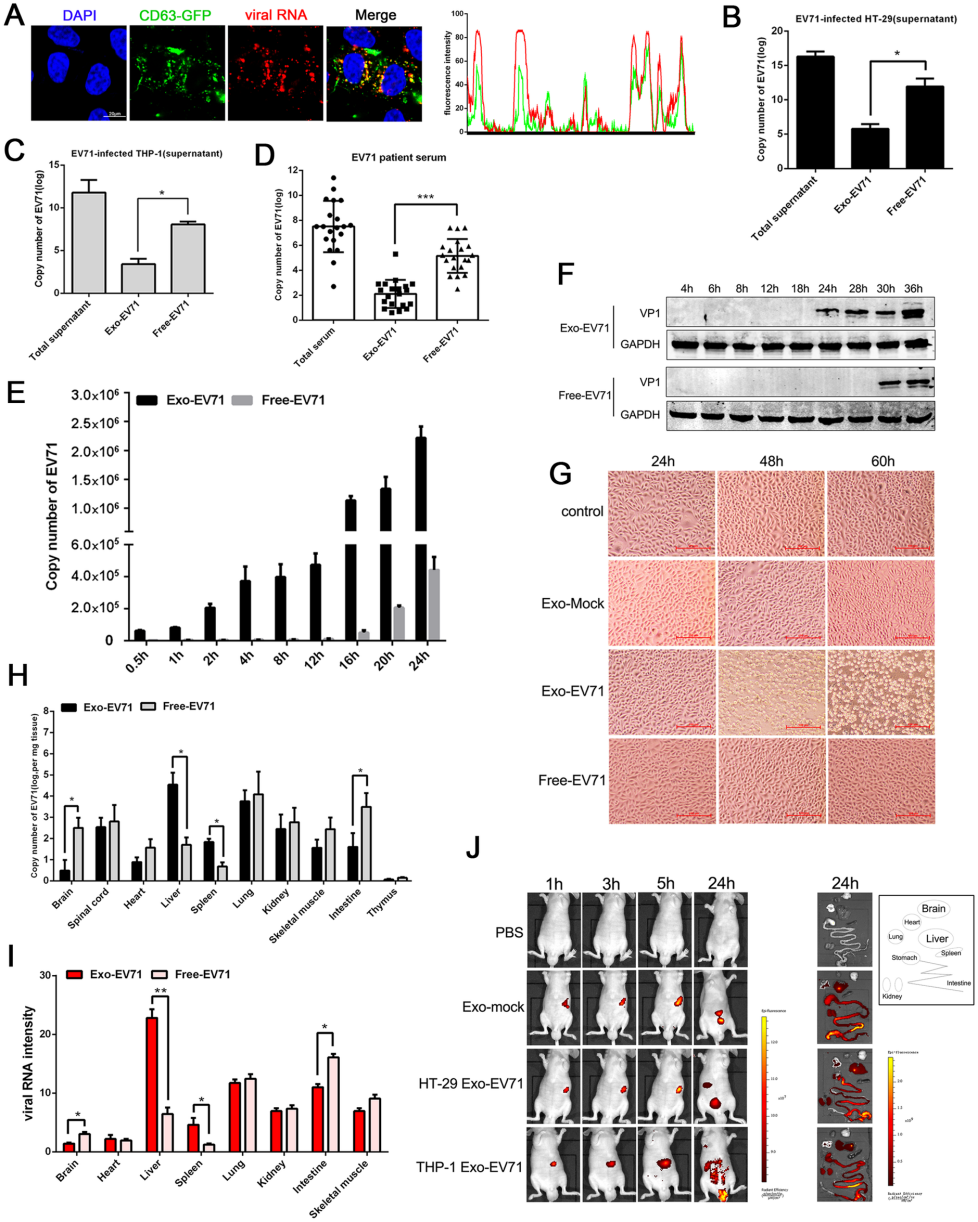
Characterization of infectivity of exosomal EV71 and free virus *in vitro* and *in vivo*. (A) Confocal co-localization analysis of exosomal marker CD63-GFP (green) and viral RNA (red). HT-29 cells were infected with EV71 at a 0.05 TCID_50_ for 24h after being transfected with CD63-GFP expression plasmid. Nuclei were stained with DAPI (blue). Right graph: fluorescence intensity of CD63-GFP (green) and viral RNA (red) in the regions delineated by a white line through ImageJ software. Bar = 20 μm. (B, C and D) Total RNA was extracted from total culture supernatants of EV71-infected THP-1 (A) and HT-29 (B) cells, as well as EV71-infected patient sera (D), flow through samples (free EV71) and immuno-magnetic CD63 selected exosomes (Exo-EV71) and analyzed for EV71 RNA content by quantitative real-time PCR. Data are shown as mean±SEM of three independent experiments. (E) Real-time PCR data for the comparison of EV71 RNA expression level in L929 cells at various time points. The cells were treated with an equal copy number of viral RNA from Exo-EV71 RNA and free EV71 virus. Data are shown as mean±SEM of three independent experiments. (F) Western blot of EV71 VP1 protein expression in recipient L929 cells after be treated with an equal copy number of viral RNA of Exo-EV71 and free EV71 virus. (G) Light microscopic images of L929 cells infected with Exo-EV71 RNA or free EV71 virus. Scale bar = 100μm. (H and I) An equal copy number of Exo-EV71 RNA and free EV71 was injected into tail veins of 4-week-old male NOD/SCID/IL2Rγ null mice (NSG, n = 4). Virus titers were determined in various organ tissues 24h after injection by real-time PCR (H) and fluorescence *in situ* hybridization(I). Data are shown as mean±SEM. (J) After intravenous injection of Exo-EV71 in nude mice, the red-fluorescence of whole animal or various organs was acquired at the indicated time points by IVIS spectrum. Radiant efficiency was measured using Living Image 3.1 software. (*p<0.05, **p<0.01, ***p<0.001).

We observed that exosomes isolated from a number of cell lines infected with EV71 contained intra-vesicle EV71 RNA (Exo-EV71 RNA) but not EV71 virus since no viral structural (VP1, VP0, VP2) or non-structural proteins (3AB, 3A, 3C, 3D) were identified in the exosome preparations (S2A Fig). The exosome-associated EV71 RNA was shown resistant to RNase H degradation, but completely degraded in the presence of detergent (S2B Fig), suggesting that the EV71 RNA is intra-vesicle. In addition, imaging analysis showed that viral RNA colocalized with the green fluorescence labeled exosomal marker CD63 intracellularly (Fig 2A). This evidence demonstrated that the EV71 RNA was loaded into the exosomes before being secreted and was not associated with the vesicles externally. In addition, we quantified Exo-EV71 RNA relative to free EV71 viral RNA in a fixed total volume and found that there were significantly higher EV71 viral copy numbers in the free virus fraction as compared to that in the exosomes in both HT-29 and THP-1 cell supernatants (approximately 2:1 ratio, Fig 2B and 2C). Similar distribution pattern was also observed in exosomes isolated from EV71 patients’ sera (approximately 2.5:1 ratio, Fig 2D). When exosomes labeled with the fluorescent dye (DiI) were incubated with Hela cells, the recipient cells exhibited high uptake efficiency, as indicated by fluorescence microscopy, as shown in S2C Fig. Paraformaldehyde-fixed cells remained negative for DiI fluorescence, confirming that exosome uptake is an active process and not a passive transfer of fluorescent label.

To determine if Exo-EV71 RNA infects cells and the relative infectivity of Exo-EV71 RNA and free EV71 virus, exosomes were isolated from HT-29-infected cells and incubated with RD cells (highly susceptible to EV71). Both Northern blot and real-time PCR confirmed that the level of viral RNA of exosome-treated cells showed no significant differences from that of the cells infected with free virus particles at various time points as shown in S2D and S2E Fig. This observation suggests that Exo-EV71 RNA is as efficient as free virus in infection of susceptible cells. However, interestingly we observed that Exo-EV71 RNA efficiently entered and replicated in L929 cells, a non-permissive mouse cell line that does not express SCARB2, the main receptor for EV71, and shows no cytopathic effects when infected with EV71 [33]. The L929 cells were treated with an equal copy number of viral RNA from Exo-EV71 RNA and free EV71 virus and the cells showed not only a higher copy number of viral RNA upon Exo-EV71 treatment but also increasing EV71 RNA copies over the time (Fig 2E), suggesting that the Exo-EV71 RNA replicated in the recipient cells. In contrast, free EV71 virus replicated poorly in L929 cells with a delayed kinetics, reflecting the restriction on the viral entry. Meanwhile, the recipient L929 cells exhibited high uptake efficiency when treated with Exo-EV71, as indicated by fluorescence microscopy, compared to the free virus treatment, indicating that exosome-mediated viral RNA entry is of higher efficiency in non-permissive cells (S2F Fig). Consistently, the viral structural protein VP1 became detectable at 24h post treatment with Exo-EV71 in contrast to 30h with free EV71 virus treatment (Fig 2F). Moreover, at 48h post treatment, Exo-EV71 RNA induced apparent cytopathic effects in L929 cells, while no cytopathic effects were observed in the free EV71 virus treatment, even at 60h post treatment (Fig 2G). Based on these observations, we investigated whether exosomes from EV71-infected patients’ sera could also transmit infection independent of viral receptor. We found that the patient’s Exo-EV71 RNA infected and induced apparent cytopathic effects in L929 cells 48h after treatment (S2G Fig). Real-time PCR results showed a higher copy number of viral RNA in L929 cells treated with Exo-EV71 RNA than that treated with free-EV71 (S2H Fig). Together, these observations suggest that exosomal viral RNA can mediate productive infection of the host cells in a receptor independent manner.

To further characterize Exo-EV71 RNA and the free virus *in vivo*, an equal copy number of Exo-EV71 RNA and free EV71 was injected into tail veins of 4-week-old male NOD/SCID/IL2Rγ null mice (NSG, n = 4) and the tissue sections were examined 24h post injection for viral RNA distribution. EV71 RNA was significantly more enriched in both liver and spleen of the animals injected with Exo-EV71 RNA than animals injected with free EV71, in which a higher level of viral RNA was identified in brain and intestine (Fig 2H). These observations were confirmed by using a highly sensitive EV71 RNA-specific fluorescence *in situ* hybridization (FISH) of the tissue sections (Fig 2I and S2I Fig). *In vivo* fluorescence imaging was also performed in mice over a period of 1-24h after Exo-EV71 RNA intravenous injection and showed that strong EV71 RNA signal (red fluorescence) was detected in liver at 1-5h post injection. At 24h post injection, the signals were observed in the liver and intestine, suggesting that exosomes first accumulated in the liver and then spread to the bowel (Fig 2J).

### Requirement of the exosomal pathway for Exo-EV71 release

**Fig 3:**
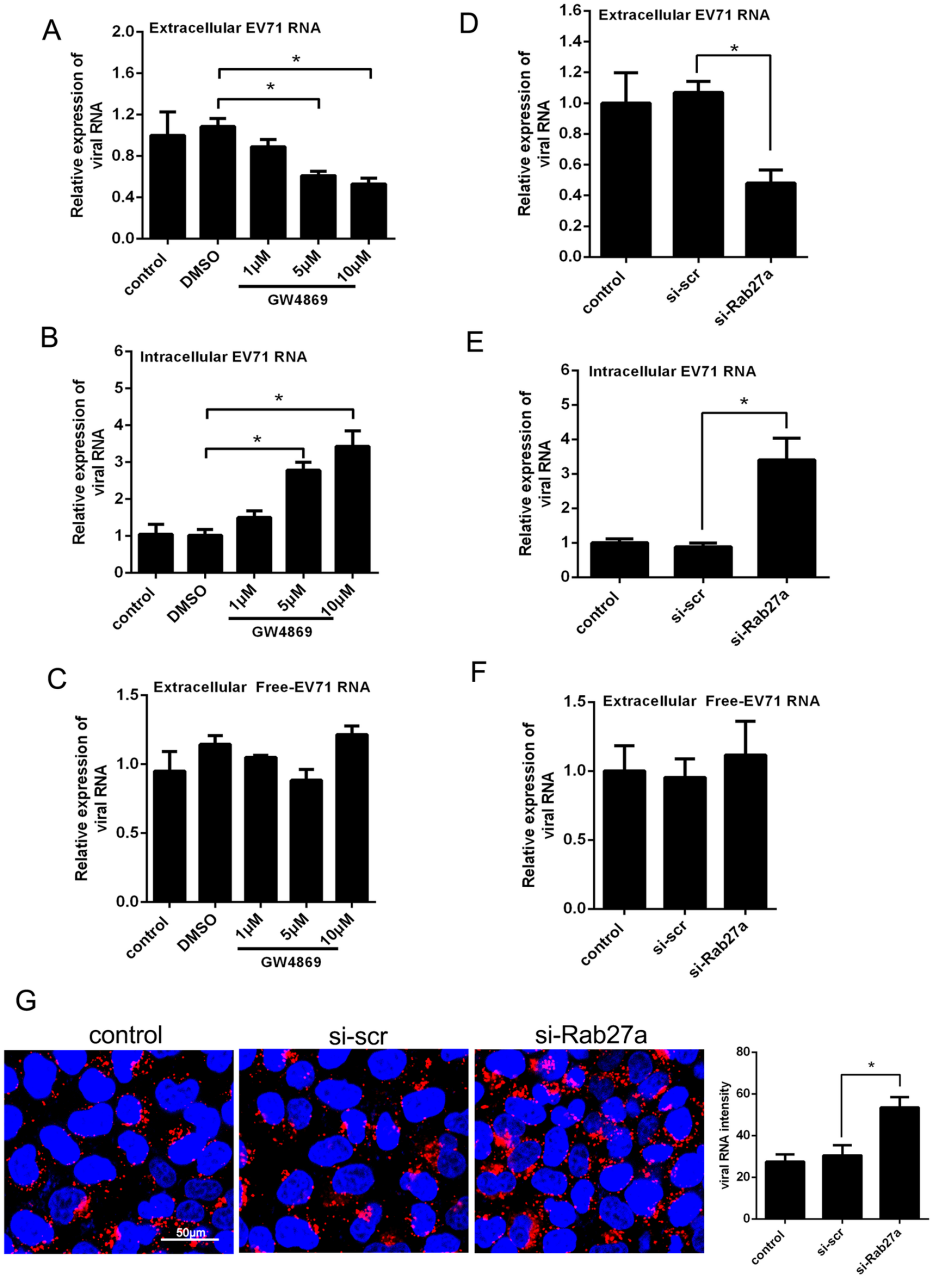
Requirement of the exosomal pathway for Exo-EV71 release. (A to C) Quantitative real-time-PCR analysis of the total extracellular (A) and intracellular (B) EV71 RNA or extracellular free EV71 RNA (C) in the infected THP-1 cells treated with GW4869 for 24h. Comparative delta-delta ct method was used for determining relative EV71 RNA levels and normalized to DMSO group. (D to F) HT-29 cells were treated with control siRNA (si-scr) or Rab27a siRNA (si-Rab27a) for 24h, followed by infection with EV71 at 0.05 TCID_50_. At 24h postinfection, extracellular (D), intracellular (E) and extracellular free EV71 (F) viral RNA levels were measured by real-time-PCR and normalized to si-scr group. (G) Fluorescence *in situ* hybridization analysis of intracellular viral RNA (red) in EV71-infected Hela cells before being tranfected with si-Rab27a and si-scr. Nuclei were stained with DAPI (blue). Bar = 50 μm. Data points presented are averaged from six different fields. All data are presented as the mean±SEM of three independent experiments. (*p<0.05).

To investigate the involvement of the exosomal pathway in Exo-EV71 release, we analyzed the effects of an inhibitor, GW4869, a drug that blocks neutral sphingomyelinase 2 (nSMase2), a crucial component in exosome secretion, on the release of Exo-EV71 RNA in EV71-infected cells. Total extracellular EV71 RNA levels were reduced to 89, 61 and 53% of the DMSO control after the cells were treated with 1, 5 and 10μM GW4869, respectively (Fig 3A). Concomitantly, intracellular EV71 RNA levels were increased by 1.5, 2.7 and 3.4-fold of the DMSO control level, respectively (Fig 3B). In contrast, the inhibitor had no significant effect on free EV71 particle release extracellularly (exosomes-depleted fraction from total supernatant) (Fig 3C). In addition, when cells were treated before EV71 infection with small interfering RNA (siRNA) against Rab27a (si-Rab27a), a Rab GTPase required for the exosome secretion [32], the EV71 RNA level in the total culture supernatant was significantly reduced by 50%, as compared to that of the cells transfected with scrambled control siRNA (si-scr) (Fig 3D). Correspondingly, the intracellular viral RNA showed a significant enrichment (~3.5-fold) as compared to the control siRNA (Fig 3E), which was supported by immunofluorescent staining of the intracellular viral RNA (Fig 3G). On the other hand, the free EV71 particle was not affected by the si-Rab27a or si-scr transfection extracellularly (Fig 3F). These results indicate that blocking exosome secretion could lead to the intracellular accumulation of EV71 viral particles without affecting EV71 RNA replication. These results suggested that the exosomal pathway is required for the release of Exo-EV71 virion.

### IFNs impaired exosome secretion induced by EV71 infection

**Fig 4:**
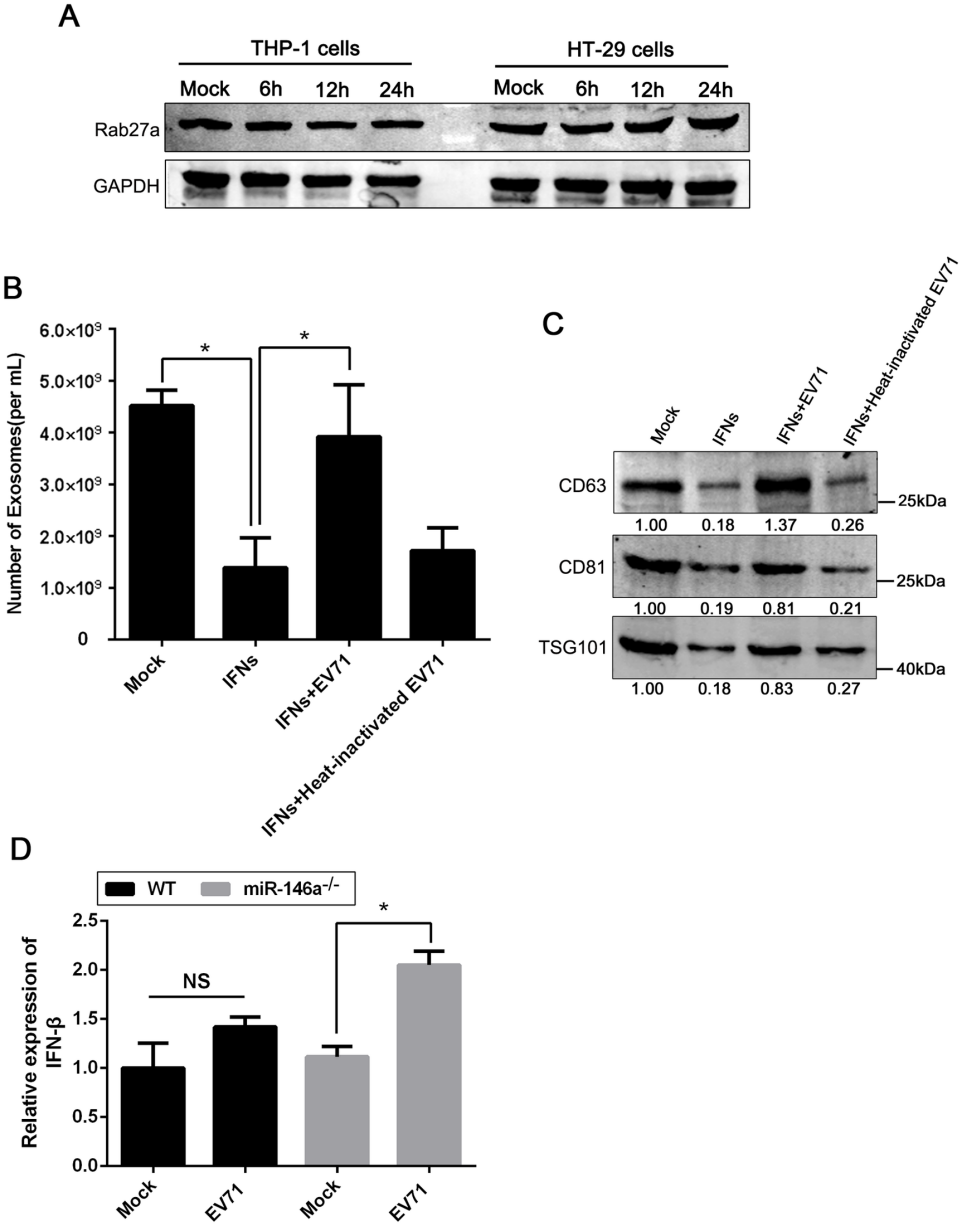
IFNs impaired exosome secretion induced by EV71 infection. (A) Western blot analysis of Rab27a was performed in cell lysates from EV71-infected THP-1 and HT-29 cells at 0.05 and 0.1 TCID_50_ respectively (B and C) NTA (B) and Western blot analysis (C) of exosomes purified by immuno-magnetic CD63 selection from cell culture supernatants of THP-1 cells treated or mock-treated with 1,000U/ml IFN-I, followed by infection with EV71 or heat-inactivated EV71 for 24h. Blots were probed for exosomal markers CD63, CD81, and TSG101 using specific antibodies. The numbers represent the relative density of the bands relative to the corresponding control. Value of mock treatment is set at 1.00 (100%). Data are shown as mean±SEM of three independent experiments. (D) The expressions of IFN-β mRNA in EV71-infected wild-type (WT) or miR-146a^-/-^ HT-29 cells by real-time-PCR. Data are shown as mean±SEM of three independent experiments. (*p<0.05).

To explore the mechanism of EV71-induced upregulation of exosome secretion, we first investigated if EV71 infection would affect Rab27a expression. As shown in Fig 4A, we did not observe a difference in the Rab27a expression between the infected and the uninfected cells, as shown by Western blot analysis, indicating that the EV71-induced upregulation of exosome production was not due to increased expression of Rab27a. A recent study reported that IFN treatment could control the secretion of the exosome [34], while EV71 infection could attenuate type I interferon response [30]. We, therefore, investigated the influence of exogenous IFN on exosome production during EV71 infection and found that the presence of IFN markedly inhibited basal level exosome production in THP-1 cells, which was partially reversed by EV71 infection (Fig 4B), consistent with the inhibitory mechanisms of EV71 on the IFN-mediated immunity [30]. The above findings were further confirmed by Western blot, showing that IFNs treatment inhibited the secretion of the classical exosome markers CD63, CD81 and TSG101 and EV71 infection could reverse the inhibition (Fig 4C).

Ho *et al*. identified that EV71 infection induced cellular miR-146a expression to inhibit IFN production through inhibition of IRAK1 and TRAF6, two key elements involved in TLR signaling and IFN production [35]. We generated a miR-146a genome knockout (GKO) HT-29 cell line (HT-29-146a^-/-^) using the Clustered Regulatory Interspaced Short Palindromic Repeat (CRISPR)/CRISPR-associated protein 9 (CAS9) technology. S3A and S3B Fig showed that our construct resulted in the excision of anticipated genomic DNA fragment, demonstrating the knockout of miR-146a gene in HT-29 cells. NTA and Western blot showed that the miR-146a knockout did not affect the yield of exosomes (S3C and S3D Fig). Interestingly, no significant alteration in exosome production was observed in HT-29-146a^-/-^ cells when infected with EV71, and no apparent changes on the expression of exosomal markers CD63, CD81 and TGS101 were detected by Western blot in the EV71-infected HT-29-146a^-/-^ cells (S3C and S3D Fig). Nevertheless, IFN-β mRNA level was significantly up-regulated in HT-29-146a^-/-^ cells infected with EV71 as compared with the mock-infected cells, whereas IFN-β mRNA exhibited a small but non-significant increase in EV71-infected WT cells (Fig 4D). These results demonstrated that IFN treatment decreased exosome secretion by EV71-infection.

### BST-2/tetherin was involved in regulating exosome secretion during EV71 infection

**Fig 5:**
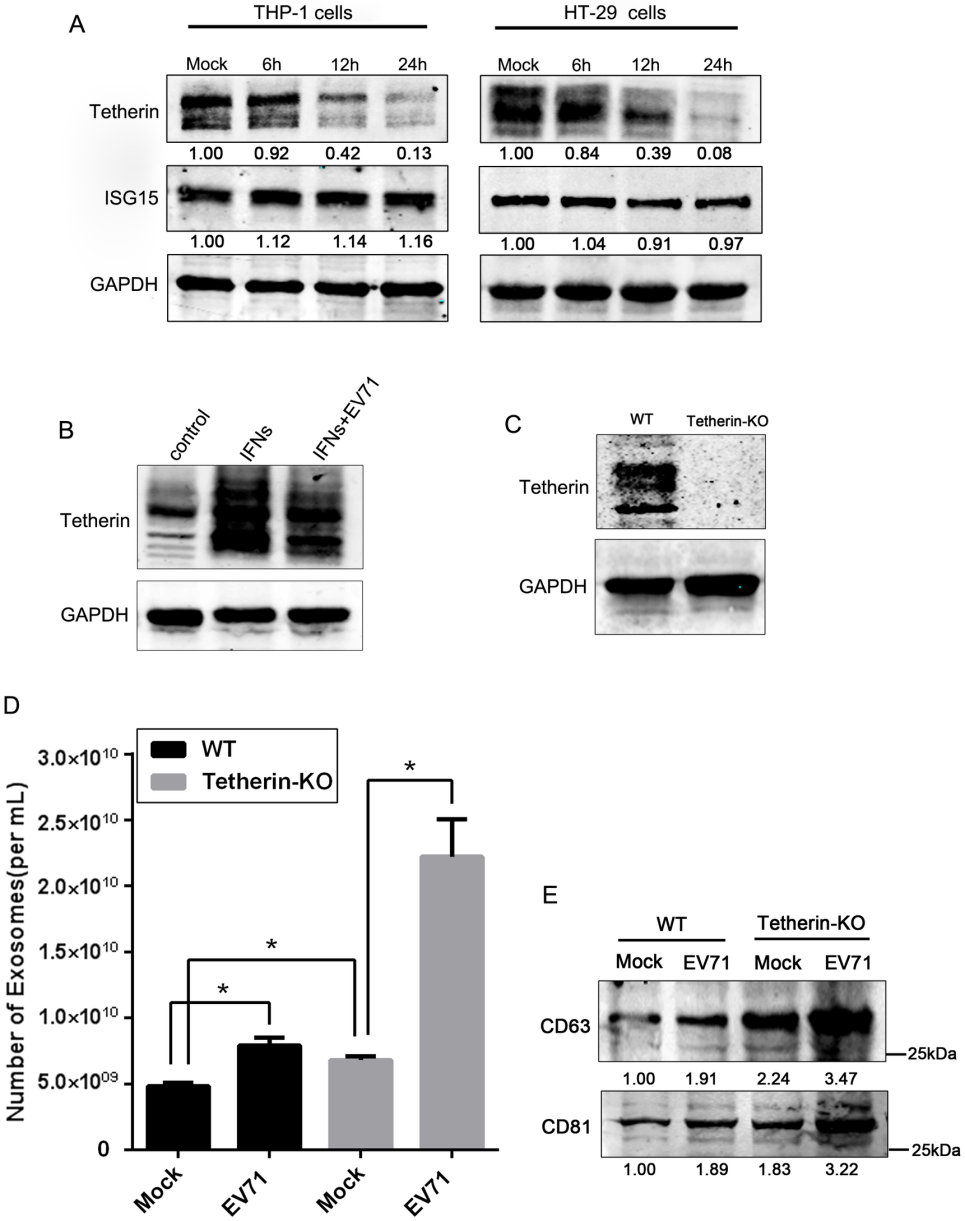
BST-2/tetherin was involved in regulating exosome secretion during EV71 infection. (A) Western blot analysis of BST-2/tetherin and ISG15 in THP-1 and HT-29 cells infected with EV71 at 0.05 and 0.1 TCID_50_ respectively for various durations as indicated. (B) Western blot analysis of BST-2/tetherin in HT-29 cells treated or mock-treated with 1,000U/ml IFN-I, followed by infection or mock infection with EV71 at 0.05 TCID_50_. (C) Western blot analysis of BST-2/tetherin expression in WT or knockout cells (Tetherin-KO) HT29 cells. (D and E) NTA (D) and Western blot analysis (E) of exosomes purified by immuno-magnetic CD63 selection of cell culture supernatants from equal numbers of WT and Tetherin-KO cells infected or mock-infected with EV71 for 24h with 0.05 TCID_50_. Blots were probed for the exosomal markers CD63 and CD81 using specific antibodies. The numbers represent the relative density of the bands relative to the corresponding mock control. Value of mock treatment is set at 1.00 (100%). Data are shown as mean±SEM of three independent experiments. (*p<0.05).

ISG15 and BST-2/tetherin, two important IFN-stimulated gene factors (ISGs), were recently reported to control exosome secretion [34, 36]. Western blot revealed decreased expression of BST-2/tetherin, but not ISG15, in EV71-infected THP-1 and HT-29 cells at 12 and 24h post infection (Fig 5A). In addition, BST-2/tetherin expression was upregulated by IFNs, which was repressed by EV71 infection (Fig 5B), suggesting that EV71 suppression of BST-2/tetherin expression was dependent on IFNs treatment.

To study the role of BST-2/tetherin in exosome secretion upon EV71 infection, we knocked out the BST-2/tetherin gene in HT-29 cells by CRISPR/Cas9-mediated genome editing. Clonal cell lines were analyzed by Western blot and showed that tetherin was no longer expressed in tetherin-KO cells (Tetherin-KO) (Fig 5C). NTA quantification revealed a ~3.2-fold increase in exosome secretion in EV71-infected Tetherin-KO cells as compared with that in the mock control (Fig 5D). This observation was further confirmed by Western blot analysis of exosome markers in the culture supernatant of WT and Tetherin-KO cells, infected or mock-infected with EV71. Although the knockout increased the basal level exosome secretion, the EV71 infection induced a larger increase of exosome secretion in Tetherin-KO than in the WT cells, as indicated by both CD63 and CD81 expression (Fig 5E). Together, our gain- and loss-of-function experiments strongly support the role of BST-2/tetherin in the negative regulation of exosome secretion during EV71 infection.

### EV71 infection induced preferential exosomal sorting of miR-146a and exosomal miR-146a could be transferred to recipient cells

**Fig 6:**
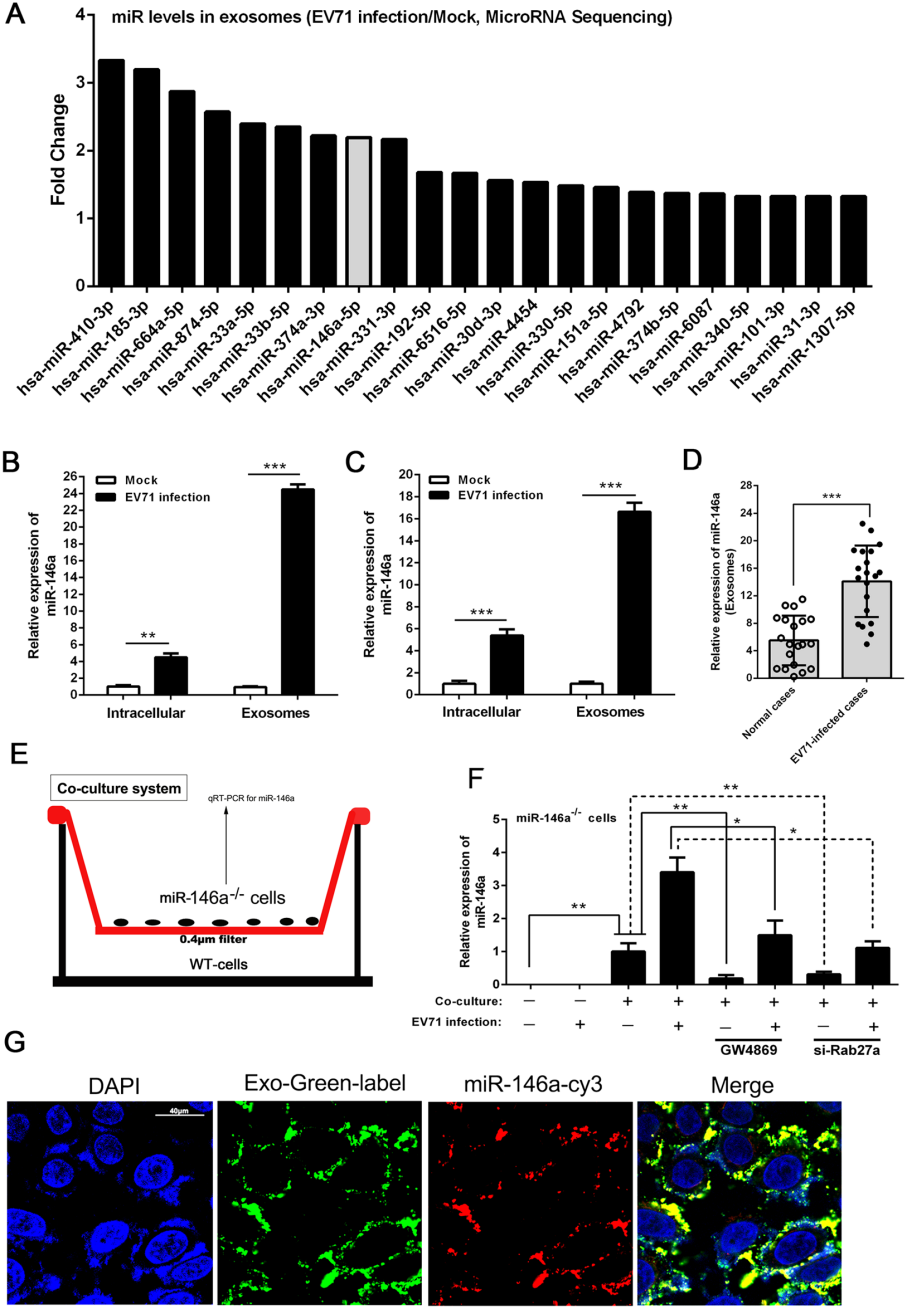
EV71-induced exosomal sorting of miR-146a was transferable to recipient cells. (A) Composition of small RNAs in exosomes derived from HT-29 cells with or without EV71 infection. 120μg purified exosomes was used. Data show the fold-changes in EV71-infected versus mock. (B to D) Real-time PCR analysis of miR-146a expression in intracellular compartments or purified exosomes of THP-1 (B) and Hela (C) cells with(0.05 TCID_50_) or without EV71 infection for 24h, as well as in exosomes isolated from sera of EV71 infected patients (D). Exogenous cel-miR-39 was used for normalization of exosomal miRNA. All reactions were run in triplicate and the analysis was carried out using 2^-ΔΔCt^ method. (E) Schematic presentation of the transwell co-culture with miR-146a^-/-^ HT-29 cells in the top well and WT HT-29 cells in the bottom well. A porous (0.4 μm) membrane allows transfer of exosomes but precludes direct cell-cell contact. (F) Real-time PCR analysis of miR-146a in the co-culture system with or without 24h EV71 infection. 10μM GW4869 or 100nM Rab27a siRNA(si-Rab27a)were used to treat donor WT cells to inhibit exosome formation. Data were shown as mean±SEM of three independent experiments. (G) Exosomal miR-146a mimic-cy3 internalization by miR-146a^-/-^ HT-29 cells. Purified Exo-Green-labeled exosomes secreted by WT HT-29 cells transfected with cy3-labeled miR-146a were incubated with miR-146a^-/-^ HT-29 cells grown on chamber slides. Fluorescent and phase contrast images were captured at 24h. Bar = 40μm. Data are shown as mean±SEM of three independent experiments. (*p<0.05, **p<0.01, ***p<0.001).

Exosomes can transfer functionally active miRNAs between cells and thereby modify or reprogram the biological activities of target cells [3, 37]. We performed a comprehensive miRNA profiling in exosomes from EV71-infected HT-29 cells by deep RNA-sequencing (RNA-seq). Based on the significance of variation as indicated by ANOVA analysis, 22 miRNAs were identified to be enriched in exosomes of EV71-infected cells, as compared to those in mock-infected cells (Fig 6A, and S1 Table). We analyzed these 22 miRNAs by quantitative real-time PCR and confirmed the up-regulation of those miRNAs in EV71-infected HT-29 cells versus those from mock-infected cells though the degrees of enrichment were not entirely consistent with the RNA-seq results (S4A Fig). To investigate the roles of these miRNAs during viral replication, we inhibited individual miRNAs with specific inhibitors before EV71 infection. Western blot analysis on viral protein VP1 showed that all miRNAs except miR-146a-5p exhibited no effect on VP1 expression (S4B Fig). Inhibition of miR-146a-5p almost completely blocked VP1 expression, suggesting its specific inhibition of EV71 replication. Therefore, subsequent study was focused on miR-146a. Using a standard real-time PCR-base quantification to confirm the RNA-seq data, we observed that upon EV71 infection, the total miR-146a expression increased by 4- and 5-fold at 24hpi in THP-1 and Hela cells as compared to the mock cellular levels of miR-146a in the respective cells. Interestingly, miR-146a was enriched selectively in exosomes by 24-fold in THP-1 cells and 17-fold in Hela cells, respectively, as compared to the exosomes from mock-infected cells (Fig 6B and 6C). We also determined the copy numbers of miR-146a per exosome using a standard real-time PCR-base absolute quantification and observed that EV71 infection increased miR-146a 13-fold in Hela cells (S4C Fig) and 20-fold in THP-1 cells on a per exosome basis (S4D Fig). We next compared exosomal miRNA levels in EV71-infected patient serum samples and observed significantly higher serum exosomal miR-146a in EV71-infected cases (sample = 20) than the normal individuals (sample = 20) (Fig 6D), supporting our observations in cells.

To determine if exosomal miR-146a could be transferred to and function in target cells, we conducted the study using WT HT-29 cells as donor and HT-29-146a^-/-^ cells as recipient in transwell chambers with 0.4μm pore size that is permeable to exosomes but not to the cells (Fig 6E). As shown in Fig 6F, a significant amount of miR-146a was detected in miR-146a^-/-^ cells co-cultured with the infected WT cells. When the donor cells were infected with EV71, the amount of miR-146a in HT-29-146a^-/-^ cells was significantly elevated. Treatment of the donor cells (WT) with GW4869 or si-Rab27a significantly inhibited the transfer of miR-146a to the HT-29-146a^-/-^ cells, suggesting that miR-146a transfer to HT-29-146a^-/-^ cells was mediated by exosomes. In order to determine if the transferred miR-146a was indeed exosome-associated, we transfected WT cells with cy3-labeled miR-146a mimics, isolated the exosomes and then labeled the exosomes with green fluorescence. The exosomes were then used to incubate with HT-29-146a^-/-^ cells. After 24h incubation with the labeled exosomes, the cy3 fluorescence was observed in >90% of the recipient cells, in which it was co-localized with the green fluorescence-labeled exosomal membranes (Fig 6G), suggesting that miR-146a was packaged in the exosomes. Together, these data showed that miR-146a can be transferred between cells by exosomes.

### Exosomal miR-146a functioned in recipient cells

**Fig 7:**
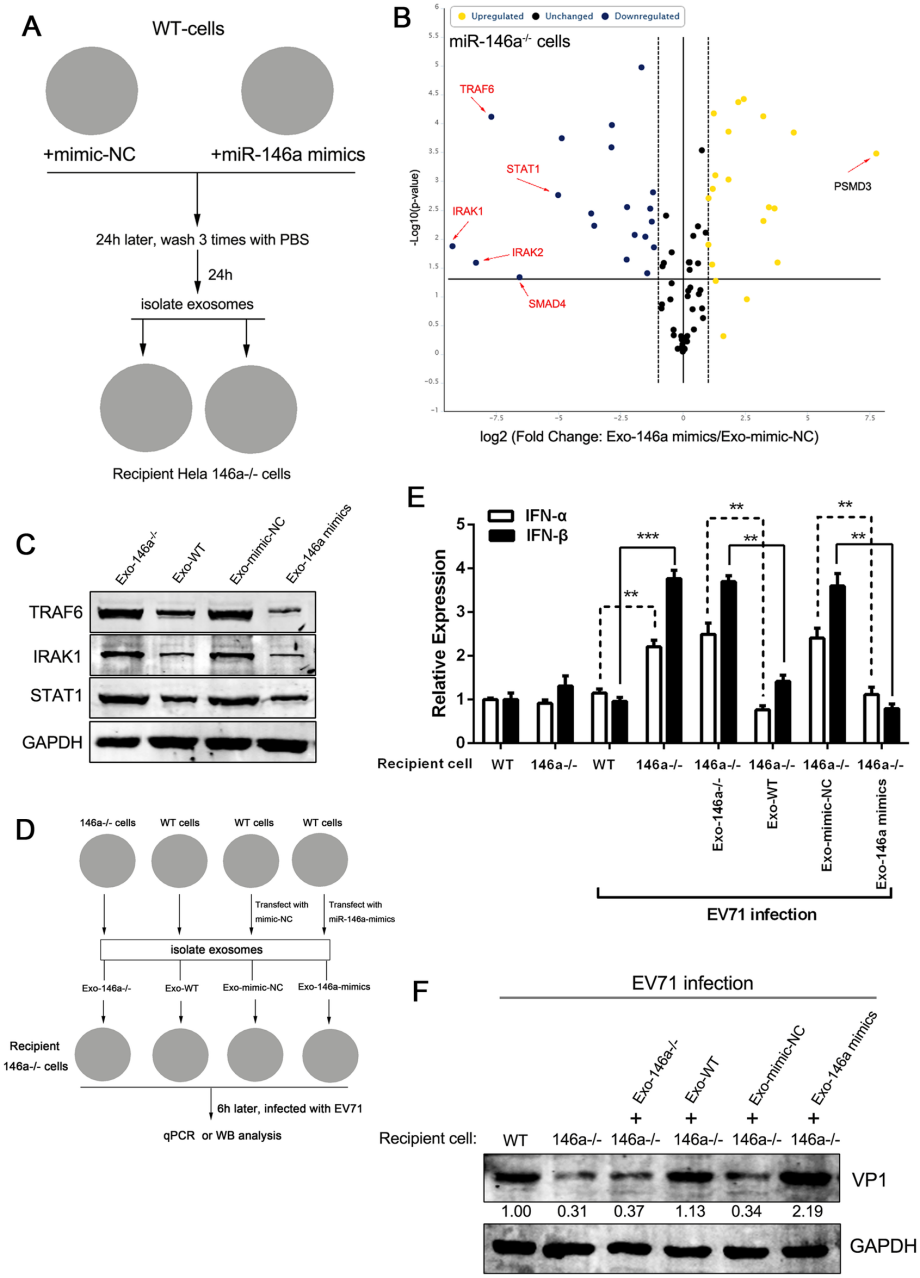
Exosomal miR-146a functions in recipient cells. (A) Schematic presentation of miR-146a mimics transfer experiment. (B) miR-146a^-/-^ HT-29 cells were incubated with 60μg purified miR-146a mimics-containing exosomes for 24h and miR-146a in miR-146a^-/-^ HT-29 cells was examined by real-time RT-PCR assay using human miR-146a Targets RT^2^ Profiler PCR Array. (C) Western blot analysis of TRAF6, IRAK1 and STAT1 in miR-146a^-/-^ HT-29 cells incubated with 60μg exosomes purified from WT or miR-146a^-/-^ HT-29 cells transfected with miR-146a mimics or negative control (NC). (D) Schematic presentation of exosome transfer experiments with exosomes isolated from various sources. (E) Quantitative real-time-PCR analysis of IFN-α and IFN-β mRNA levels in the recipient cells that were infected with or without EV71. Data are shown as mean±SEM of three independent experiments. (F) Western blot analysis of EV71 VP1 protein expression in recipient cells of the exosome transfer experiment. The numbers represent the relative density of the bands in comparison to the corresponding control normalized to GAPDH. Value of mock recipient WT cells is set at 1.00 (100%).

To investigate whether the transferred exosomal miR-146a is biologically active in the recipient cells, we isolated exosomes from WT HT-29 cells transfected with either miR-146a mimics or negative control mimics (mimic-NC) and transferred them to HT-29 miR-146a^-/-^ cells (Fig 7A). 24h after transfer, total RNA was isolated from miR-146a^-/-^ cells and analyzed by real-time RT-PCR using human miR-146a Targets RT^2^ Profiler PCR Array profiles that contain 84 genes either proven or predicted to be regulated by hsa-miR-146a-5p. As shown in Fig 7B and S2 Table, we observed that five mRNAs, TRAF6, STAT1, IRAK1, IRAK2 and SMAD4, showed the most reduction in HT-29 miR-146a^-/-^ cells treated with miR-146a mimics-containing exosomes. Moreover, the transfer of exosomal miR-146a mimics resulted in downregulated expression of respective target proteins in HT-29 miR-146a^-/-^ cells, while exosomes that carried mimic-NC caused no changes in target protein expression (Fig 7C). Meanwhile, the protein expressions of TRAF6, IRAK1 and STAT1 were also repressed in miR-146a^-/-^ cells treated with exosomes isolated from WT but not miR-146a^-/-^ cells (Fig 7C), though to a less extent, suggesting that the repression was miR-146a specific. We further analyzed whether the transferred exosomal miR-146a could affect recipient cell response to EV71 infection by incubating HT-29 miR-146a^-/-^ cells with 60μg exosomes isolated from WT (Exo-WT), miR-146a^-/-^ (Exo-146a^-/-^), or mimics-transfected (Exo-146a mimics) HT-29 cells, followed by EV71 infection for 24h (Fig 7D). Consistent with the previously reported roles of miR-146a in facilitating viral pathogenesis by suppressing type I interferon production during EV71 infection [35], HT-29-miR-146a^-/-^ cells treated with either Exo-WT or Exo-146a mimics expressed significantly lower IFN-α/β upon EV71 infection than the cells treated with Exo-146a^-/-^ (Fig 7E). We also observed that miR-146a knockout (146a^-/-^) could restore IFN-α/β expression, as compared to WT cells infected with EV71 (Fig 7E). Further analysis by Western blot showed that EV71 structural protein VP1 expression was upregulated by the Exo-WT or Exo-146a mimics in HT-29-miR-146a^-/-^ cells, as compared to the cells treated with Exo-146a^-/-^ and Exo-mimic-NC (Fig 7F). These results indicated that miR-146a could be transferred to neighboring uninfected cells via exosomes and suppress type I interferon production, thus facilitating viral replication.

Report showed that sorting of specific miRNAs into exosomes is controlled by Ago2 protein, a key component of the RISC that can directly degrade mRNA by slicing [38]. As a component of miRNA effector complexes, Ago2 also associates with the interacting partner GW182, which has been shown to associate with MVBs and be secreted by the exosomes [39]. To analyze whether Ago2 and GW182 interact with miR-146a in exosomes, we first performed co-immuno precipitation study and showed that Ago2 and GW182 formed complex within the exosomes (S5A Fig). To investigate whether Ago2 and GW182 associate with miR-146a in exosomes, RNA-protein complexes were immunoprecipitated with antibodies specific to Ago2 or GW182 and RNA was isolated from the immune complex and analyzed by Northern blot. We found that Ago2-GW182 complex was associated with miR-146a (S5B Fig). By co-immunoprecipitation, we also observed that Ago2-GW182 complex was associated with EV71 RNA (S5C Fig). A previous study reported that Ago2 promoted EV71 virus replication by interacting with the viral IRES [8]. These observations suggest that by complexing with Ago2-GW182, EV71 may stabilize its genomic RNA while miR-146a suppresses IFN-β expression through a preformed RISC immediately after the virus enters the host cells in a coordinate manner.

### Exosomal miR-146a was required for Exo-EV71 RNA replication in recipient cells

**Fig 8:**
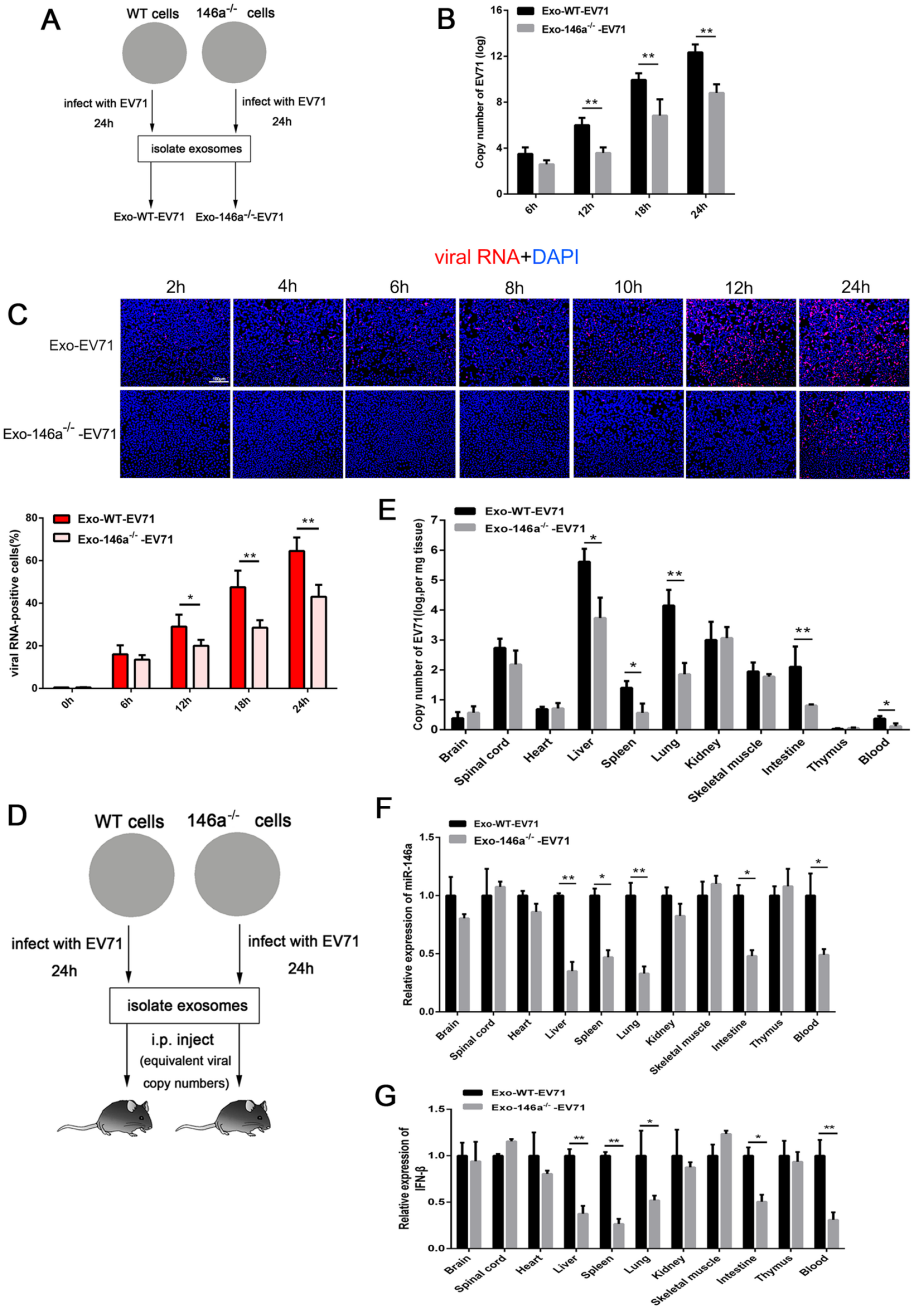
Exosomal miR-146a was required for Exo-EV71 RNA replication in recipient cells. (A) Schematic presentation for exosomes isolated from EV71-infected WT (Exo-WT-EV71) and miR-146a^-/-^ (Exo-146a^-/—^EV71) HT-29 cells. (B) Quantitative real-time-PCR analysis of EV71 RNA in Hela cells treated with Exo-WT-EV71 or Exo-146a^-/—^EV71 for indicated time. Data are shown as mean±SEM of three independent experiments. (C) Viral RNA expression determined by fluorescence *in situ* hybridization (FISH) after Hela cells were treated with Exo-WT-EV71 or Exo-146a^-/—^EV71 for indicated time. Nuclei are identified by DAPI staining. Data points presented are averaged from six different fields. (D) Schematic presentation of the *in vivo* experiment. (E, F and G) An equal copy number of Exo-WT-EV71 and Exo-146a^-/—^EV71 was injected into tail veins of 4-week-old male NOD/SCID/IL2Rγ null mice (NSG, n = 4). Virus titers (E), miR-146a (F) or IFN-β(G) were determined in various tissues by real-time PCR. (*p<0.05, **p<0.01).

To address the biological roles of the co-packaging of viral RNA and miR-146a, we investigated if exosomal miR-146a would facilitate Exo-EV71 RNA infection of a new host. Exosomes isolated from EV71-infected HT-29 WT (Exo-WT-EV71) and HT-29-miR-146a^-/-^ (Exo-146a^-/—^EV71) cells were incubated with Hela cells (Fig 8A) and the viral RNA copy number was determined in Hela cells. Results showed a significantly higher copy number of viral RNA (2–3 logs) in the cells treated with Exo-WT-EV71 at 12h, 18h and 24h than the cells treated with an equal copy number of Exo-146a^-/—^EV71 (Fig 8B). This observation was also confirmed by fluorescence *in situ* hybridization (FISH) for viral RNA. The percentage of viral RNA-positive Hela cells treated with Exo-WT-EV71 was significantly higher at 12h, 18h and 24h than the cells treated with Exo-146a^-/—^EV71 (Fig 8C). *In vivo* study was also performed to further investigate the role of exosomal miR-146a in EV71 infection by injecting NOD/SCID/IL2Rγ null mice with exosomes isolated from EV71-infected HT-29-WT and HT-29-miR-146a^-/-^ cells (Fig 8D). The dosage of the exosomes contained equal copy numbers of viral RNA to ensure a comparable viral inoculum. After 24h, the mice were sacrificed for tissue sections and analyzed for viral RNA distribution. We found that liver, lung, intestine and blood had significantly higher EV71 viral RNA in animals injected with Exo-WT-EV71 than the animals treated with Exo-146a^-/—^EV71. Other organs showed comparable viral RNA levels in both treatment groups (Fig 8E). Correspondingly, the miR-146a and IFN-β level were significantly lower in liver, lung, intestine and blood upon Exo-146a^-/—^EV71 injection as compared to the animals treated with Exo-WT-EV71, but no significant differences in other organs (Fig 8F and 8G). These observations are consistent with the viral RNA levels in the respective organs, suggesting that the higher miR-146a expression in these organs facilitates EV71 RNA replication by downregulating IFN response.

## Discussion

Exosomes are membrane-enclosed vesicles actively released into the extracellular milieu, whose content reflects the physiological/pathological state of the cells from which they originate [1–3]. Although exosomes and their contribution to replication and pathogenesis of viruses remain largely unexplored, a number of RNA viruses have been investigated, such as HIV-1, HTLV-1, HCV and Dengue virus [8–11, 40, 41]. In the current study, we showed that EV71 infection upregulated exosome secretion and resulted in differential packaging of the viral genomic RNA and miR-146a. We provided evidence that exosomal miR-146a can be transferred to and suppress type I interferon expression in the recipient cell, thus facilitating the viral replication.

Due to the similarity of EV71 virion and exosomes in their buoyant densities and sedimentation velocities [3, 42], the traditional ultracentrifugation and sucrose gradient isolation method is insufficient for isolating pure exosomes free of viral and cellular contamination; thus, we optimized the CD63 immuno-magnetic isolation method to purify EV71-associated exosomes without carryover of free virus, thereby ensuring that the EV71 RNA was intra-exosomal. A recent study showed that EV71 infection could be transmitted by exosomes and established a productive infection [31]. However, the viral structural proteins, including the VP1, which were detected in the exosomes isolated from EV71-infected RD cells [31], were not detected in exosomes under our experimental conditions. The discrepancy is likely due to our more stringent two-step purification methods since the ultracentrifugation method alone is known to have exosome preparation contaminated with large amount of protein complexes [37, 43]. Moreover, we found that exosome-mediated transmission of EV71 could resist RNase degradation, which was similar to a previous report on HCV [40]. The observation that exosomal EV71 RNA resists RNase degradation may also have biological implication. Since EV71 is transmitted via fecal-oral route, the exosomal EV71 RNA would survive better from digestive enzymes enriched in the oral cavity than naked virion. In addition, the exosomal EV71 RNA may avoid immune recognition and activation during transmission [12]. Notably, recent studies showed that hepatitis A virus (HAV), an important cause of enterically transmitted hepatitis in humans, was released from cells cloaked in host-derived membranes, thereby protecting the virion from antibody-mediated neutralization and probably promoting virus spread. These “quasi-enveloped” (eHAV) virions are similar in size and density to exosomes and have specific infectivity similar to naked, nonenveloped HAV particles [44, 45]. Moreover, Coxsackievirus B, poliovirus, and many other members of the enterovirus were also released within extracellular vesicles before cell lysis, often with many capsids packaged within a single vesicle [46–48]. These studies, including our findings, strongly suggest that cellular egress of these classically nonenveloped viruses in membrane-bound vesicles has blurred the distinction between enveloped and nonenveloped viruses and has important implications for pathogenesis [49].

Although Exo-EV71 RNA and free EV71 virus exhibited similar entry efficiency to permissive cells (S2C and S2D Fig), Exo-EV71 RNA entry into non-permissive L929 cells was much more efficient than free EV71 virion. Previous studies showed that SCARB2 and PSGL-1 serve as cellular receptors for EV71 infection [33, 50, 51]. Since L929 cells do not express SCARB2 and PSGL-1 [52], our observations suggest that exosomal viral RNA could overcome the restriction of the entry receptor and mediate productive infection of the host cells in a receptor independent manner. It is plausible that by packaging viral genomic RNA into exosomes, the virus may have a broader host range because the infection is not restricted by the presence of a cellular receptor. Our *in vivo* study found that EV71 RNA enriched in both liver and spleen when animals were injected with Exo-EV71 RNA, distinct from the animals injected with free EV71, in which high level of viral RNA was identified in brain and intestine. Although previous studies have suggested that abundant SCARB2 in various organs is the basis of systemic EV71 infection and SCARB2 functions as a receptor for EV71 in humans but not mice, there was no clear correlation between mouse SCARB2 and EV71 antigen distribution in the mouse model [52–54]. Nevertheless, the EV71-induced lesions seen in the mouse model resembled the pathological changes seen in human tissues [53]. Autopsy studies have revealed that SCARB2 was highly positive in the cerebellum, brainstem, spinal cord, lung, and glandular epithelial cells of the intestine, but weakly expressed or negative in skeletal muscle, liver, kidney, spleen and pancreas [55]. Moreover, EV71 is a neurotropic pathogen and causes severe pathological lesions in the CNS [20, 21, 55]. Therefore, we speculate that the possible pathway for this organ distribution is that free EV71 infects brain and intestine tissues because of their high SCARB2 expression while exosome-mediated viral RNA is enriched in liver and spleen since both do not express the receptor. In addition, the liver is known to be an immunologic organ playing key roles in host defense against pathogens [56]. The liver is enriched with cells key to innate immunity, such as macrophages, natural killer and natural killer T etc. The accumulation of exosomal EV71 RNA and miR-146a in the liver may be a result of antagonizing the innate IFN immune response in mononuclear phagocyte systems.

**Fig 9:**
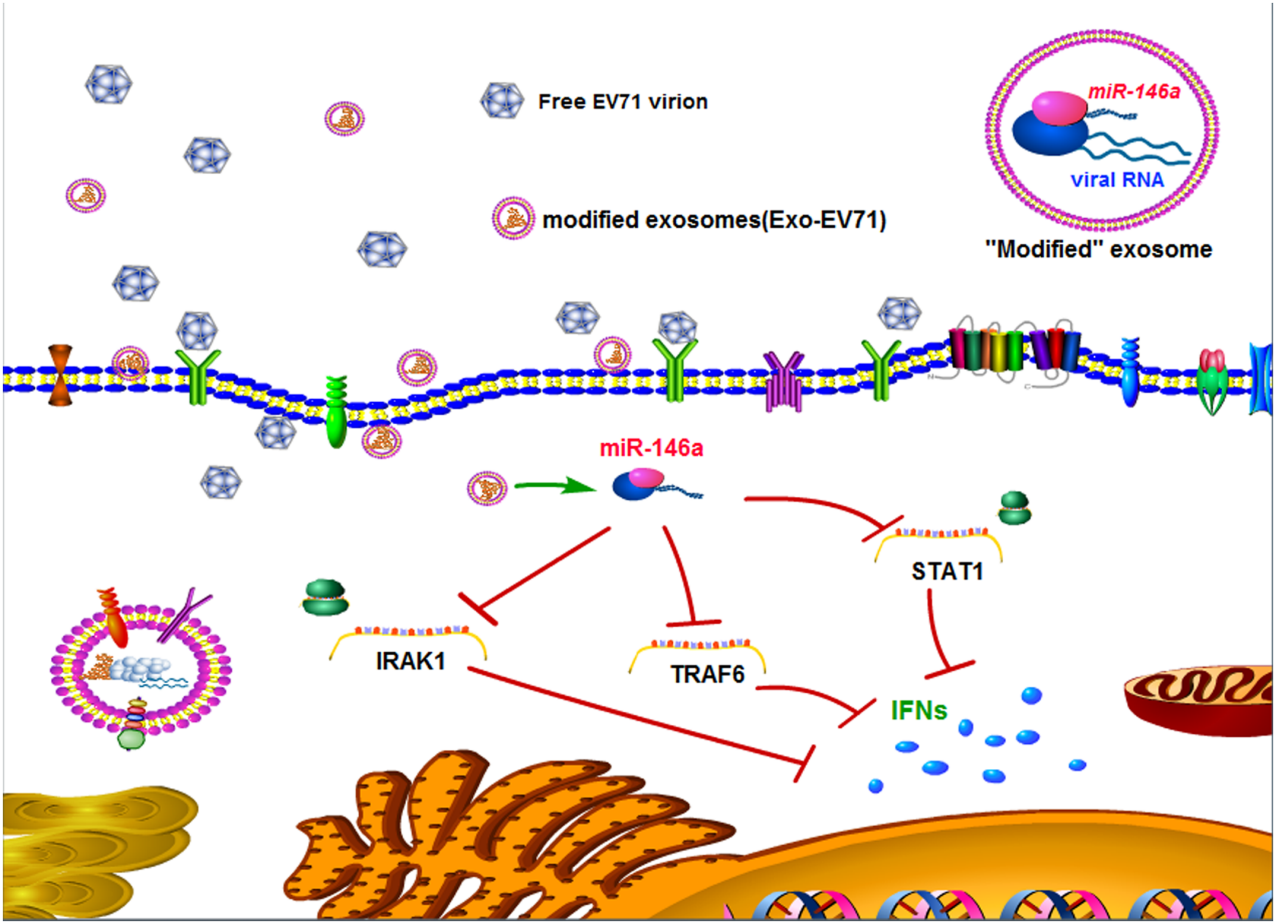
Model for the regulatory role of exosomal miR-146a in enterovirus 71 infection. The exosomal EV71 RNA could be transferred to and replicate in a new target cell while the exosomal miR-146a suppression of IRAK1, TRAF6 and STAT1 and inhibition of IFN production in the target cell, thus facilitating the viral replication. Moreover, the exosomes contained replication-competent viral RNA in complex with miR-146a, Ago2, and GW182 and could mediate EV71 transmission independent of virus-specific receptor.

Apart from viral RNAs, exosomes can deliver nucleic acid through their cargos of selectively packaged cellular miRNAs, which are the most abundant RNA in exosomes [3, 14, 17, 18, 37]. Through deep RNA-sequencing and real-time PCR, we confirmed that EV71-induced miR-146a was enriched in exosomes and transferable to new host cells, resulting in elevation of miR-146a level in the recipient cells. Earlier study found that EV71 infection induced cellular miR-146a expression to inhibit IFN production mediated by IRAK1 and TRAF6, two key elements involved in TLR signaling and IFN production, and evade host immune defense [35]. Our results confirmed that cells that received exosomal miR-146a could block the signaling for type I interferon production, facilitating viral pathogenesis. By co-delivering miR-146a, Exo-EV71 RNA may gain transmission advantages by suppressing IFN response immediately after viral entry, needing minimal viral inoculum and rapidly establishing productive infection (Fig 9). In fact, previous study demonstrated that exosomes released from HCV-infected cells contained HCV RNA, which could be successfully transferred to non-permissive plasmacytoid DCs (pDCs) and subsequently triggered the production of type I IFN [57]. Notably, Feng *et al* reported that the eHAV, not viral RNA exosomes, was responsible for interferon induction in pDCs activation, which did not require virus replication and was associated with efficient eHAV uptake [58]. In addition, IFN-induced antiviral response could be transmitted from nonpermissive liver nonparenchymal cells (LNPCs) to HBV-infected hepatocytes via exosomes and thus restored the antiviral state in hepatocytes [59]. It is clear that in viral infections, exosomes play a dual role in the modulation of the immune system, both serving as a host program to induce innate immunity and as a viral strategy to evade those same responses.

Additionally, the association of Ago2 and GW182 with both EV71 RNA and miR-146a in the exosomes will not only stabilize the viral genomic RNA, directly facilitating its replication [60], but also allow the miR-146a ready to act on its cognates immediately upon entry into recipient cells. Similarly, a recent study reported that exosomes derived from HCV-infected individuals or HCV-infected Huh7.5 cell supernatants have been found to contain HCV viral RNA, in association with Ago2, HSP90, and miR-122 [40]. It was known that the binding of miR-122 to the 5’UTR of HCV genomic RNA is critical for viral replication by moderately stimulating viral protein translation and, in concert with AGO, by stabilizing and protecting the uncapped HCV RNA genome from degradation [41, 61–64]. The presence of host proteins within virus-containing exosomes is a smart strategy by the virus to ensure effective replication once in the cytoplasm [65].

To date, information on exosome secretion induced by virus infection remains limited. HIV Nef protein was reported to increase the production of exosomes and was released through exosomes [11, 41]. Hurwitz *et al*. demonstrated that the LMP1, an Epstein-Barr virus (EBV) encoded oncoprotein, enhanced exosome secretion in a variety of cellular models relevant to EBV pathogenesis [66]. In our study, we observed that EV71 infection upregulated exosome production in HT-29 and THP-1 cells. Of interest is that CA16 did not have any effect on exosome production. One possible explanation for the difference in exosomal secretion by EV71 and CA16 could be the structural differences between non-structural proteins that possess protease activity (2A and 3C) despite their similarity in genomes and protein functions [21]. These proteins perform multiple roles during virus infection, further influencing the pathogenicity [67–69]. For instance, those non-structural proteins contribute to the profound differences on the IFN pathway, with EV71 capable of antagonizing the innate IFN immune response through weakening the activation of the JAK-STAT pathway, whereas CA16 stimulating the JAK-STAT pathway [30, 70].

Although Rab27a regulates exosome secretion by docking MVB at the plasma membrane [32], we did not observe a difference in the Rab27a expression during EV71 infection. EV71 suppression of the JAK-STAT activation would further repress the expression of IFN-stimulated gene factors (ISGs) [70]. Recent studies reported that ISG15 and BST-2/tetherin, two important ISGs, could control exosome secretion [34, 71]. Our evidence supports the roles of BST-2/tetherin in the regulation of exosome secretion during EV71 infection. BST-2/tetherin is a transmembrane protein that localizes at the plasma membrane, as well as the membranes of multiple intracellular vesicles, including endosomes and the trans-Golgi network [36, 72]. The IFN-induced BST-2/tetherin inhibits hepatitis B virus (HBV) release from cells, and the colocalization of BST-2/tetherin and HBV particles on MVBs has been reported [73, 74]. In addition, BST-2/tetherin is highly expressed in cells of the immune system and its expression can be dramatically upregulated by interferons [74–76]. It is tempting to speculate that one of IFNs mechanisms of action may be to prevent exosome release, by upregulating tetherin expression during virus infection. By the way, EV71-mediated exosomal miR-146a may also partly affect the production of exosomes in recipient cells due to inhibition of type I interferons production, though the detail conjectures require further study for confirmation.

## Materials and methods

### Cell lines and cell cultures

Human colorectal cell line (HT-29), human cervical carcinoma cell line (Hela), human rhabdomyosarcoma cell line (RD) and the green monkey kidney cell line (Vero) were maintained in Dulbecco’s modified Eagle's medium (DMEM) containing 10% fetal bovine serum (FBS) in a 37°C humidified atmosphere of 5% CO_2_. THP-1 cells, the human monocytic cells derived from an acute monocytic leukemia patient, were cultured in RPMI-1640 medium containing 5 mM L-glutamine and 10% fetal bovine serum. All cell lines were obtained from ATCC. The concentration of human interferon (IFN-I, PEPROTECH) used for treatment was indicated in the figure legends.

### Ethics statement

All animal experimental protocols were approved by the Nanjing University Animal Care Committee and followed the ‘Guide for the Care and Use of Laboratory Animals’ published by the Chinese National Institutes of Health. The research protocols were conducted in accordance with the animal behavioral guidelines, using approved protocols from the institutional animal care committee (#2014-SR-079). Serum sample collection in this project was approved by the Ethics Committee of Jiangsu Provincial Center for Diseases Prevention and Control and written informed consent was obtained from parents or legal guardians of all children.

### Nanoparticle tracking analysis

Quantification of immuno-magnetic CD63 bead-captured exosome was determined by measuring the rate of Brownian motion using a NanoSight LM10 system, which is equipped with fast video capture and particle-tracking software (NanoSight, Amesbury, UK). Exosomes were eluted from the microbeads by ice-cold 100 mM Glycine-HCl (pH 3.0) and immediately neutralized to pH 7.4 with neutralizing buffer (1M Tris-HCl, pH8.5). Samples were diluted before analysis to between 2 ×10^8^ and 20× 10^8^ particles per ml, and the relative concentration was calculated according to the dilution factor. Data analysis was performed with NTA 2.3 software (Nanosight). Samples were analyzed using manual shutter and gain adjustments, which resulted in shutter speeds of 15 or 30 ms, with camera gains between 280 and 560. The detection threshold was kept above 2; blur: auto; minimum expected particle size: 50 nm. Each sample was analyzed four times and the counts were averaged.

### Small RNA deep sequencing analysis

Exosomes were isolated from HT-29 cells infected or mock-infected with Enterovirus 71 (EV71) as described above. Total RNA isolated from exosomes was separated by 15% agarose gels to extract the small RNA (18–30 nt). After precipitated by ethanol and centrifugal enrichment of small RNA sample, a library was prepared according to the method and process of Small RNA Sample Preparation Kit (Illumina, RS-200-0048). Deep sequencing of the exosomal miRNA profiles was performed by Annoroad Genomics with the Illumina Hiseq 2500 platform with three different exosome preparations.

### Western blot

Western blot assay was carried out as follow: cells were lysed with RIPA buffer (Santa Cruz, USA) and cleared lysate was collected by centrifugation for protein separation on 10% SDS-polyacrylamide gel. The proteins were transferred onto PVDF membranes (Millipore) and detected with respective antibodies at 4°C overnight, followed by incubation with either IRDye Fluor 680-labeled IgG or IRDye Fluor 800-labeled IgG secondary antibody (Li-Cor Bioscience). The images were scanned and quantified by densitometric analysis by Li-COR Odyssey Infrared Imager. Primary antibodies against VP1 (Millipore), CD81 (Cell Signaling Technology), IRAK1 (Santa Cruz), CD63 (Abcam), ISG15 (Abcam) and Rab27a, TRAF6, STAT1, TSG101, BST-2/Tetherin, and GAPDH (all from Proteintech), VP2, 3AB, 3C and 3D (all from Genetex) were used.

### RNA extraction, reverse transcription, and qRT-PCR analysis

Total RNA was extracted using TRIzol reagent (Life Technologies) and reverse transcribed using PrimeScript RT Master Mix for RT-PCR (TaKaRa). A total of 250 fmol of synthetic *Caenorhabditis elegans* miRNA cel-miR-39 (RiBoBio, Guangzhou, China) was added to each sample during the isolation of exosomal RNA. Quantitative real time-PCR (qRT-PCR) was performed using ABI SYBR Green Master Mix (Life Technologies) on ABI Prism 7300 Sequence Detection System. GAPDH was used for normalization of mRNA (including EV71 viral RNA quantification), and exogenous cel-miR-39 and endogenous U6 small nuclear RNA were used for normalization of exosomal and cellular miRNA, respectively. All reactions were carried out in triplicate and analysis was carried out using 2^-ΔΔCt^ method. The sequences of real-time PCR primer pairs were as follow:

EV71viral RNA:
   Forward: 5’-GCTCTATAGGAGATAGTGTGAGTAGGG-3’;
   Reverse: 5’-ATGACTGCTCACCTGCGTGTT-3’.
IFN-α:
   Forward: 5’-CTGAATGACTTGGAAGCCTG-3’;
   Reverse: 5’-ATTTCTGCTCTGACAACCTC-3’.
IFN-β:
   Forward: 5’-TAGCACTGGCTGGAATGAG-3’;
   Reverse: 5’-GTTTCGGAGGTAACCTGTAAG-3’.
GAPDH:
   Forward: 5’-TGCACCACCAACTGCTTAGC-3’;
   Reverse: 5’-GGCATGGACTGTGGTCATGAG-3’.

To determine miR-146a copy numbers, standard curves were generated using high-performance liquid chromatography (HPLC) and polyacrylamide gel electrophoresis (PAGE)-purified oligoribonucleotides (RiBo Biotech, Guangzhou, China) corresponding to miR-146a sequences. The synthetic miRNA input ranged from 1 fM to 100 pM, and the reaction products were analyzed using an ABI SYBR Green Master Mix (Life Technologies). For copy numbers of miR-146a per exosome, RNA was extracted from fixed numbers of exosomes using TRIzol after counting exosomes under NTA. The average value of miR-146a per exosome was determined as described above.

### Human miR-146a targets PCR Array

miR-146a^-/-^ HT-29 cells were incubated with purified miR-146a mimics-containing exosomes for 24h and miR-146a in miR-146a^-/-^ HT-29 cells was examined by real-time RT-PCR assay using human miR-146a Targets RT^2^ Profiler PCR Array (SuperArray Bioscience Corporation, Qiagen) was probed on an ABI 7300 system (Applied Biosystems, CA) to analyze the relative expression of 84 genes involved in hsa-miR-146a-5p target genes. Data were analyzed using the 2^-ΔΔCt^ method with online SABiosciences Data Analysis Software. All tested gene expression was normalized to the expression of an internal control gene. The fold changes as described in S2 Table.

### Determination of viral RNA copy number

EV71 Fuyang0805 strain was a kind gift from Dr. Bin Wu, Jiangsu Provincial Centers of Disease Control and propagated on Vero cells. qRT-PCR was used to determine the viral RNA copy number. Briefly, total RNA was isolated from cultured cells or mouse tissues using TRIzol reagent and then reverse transcribed using random hexamers with a reverse-transcription kit (TaKaRa). The cDNA was subjected to quantitative PCR using ABI SYBR Green Master Mix (Life Technologies) on an ABI 7300 system for 40 cycles. The primers were EV71-F (5’-AGATAGGGTGGCAGATGTAATTGAAAG-3’) and EV71-R (5’- TAGCATTTGATGATGCTCCAAT-3’). A fragment corresponding to nucleotides 2462–2635 of FY0805 was adjusted to a concentration gradient (1×10^1^ copies/μl to 1×10^8^ copies/μl) and was used as a standard to calculate the copy number of viral RNA. Semi-quantitative RT-PCR was used to confirm the results of qRT-PCR.

### Transmission electron microscopy

Exosomes isolated by positive anti-CD63 immuno-magnetic bead selection were re-suspended in PBS and examined under transmission electron microscope. Images were acquired as previously described [34].

### Generation of miR-146a and BST-2/Tetherin knockout cells line

The lentiviral vector lentiCRISPRv2, which expresses clustered regularly interspaced short palindromic repeats (CRISPR/Cas9) and guide RNA (gRNA) (AddGene 73179), the lentiviral packaging plasmid pMD2.G (AddGene 12259) and psPAX2 (AddGene 12260) were obtained from AddGene. Using the CRISPR online design tool (http://www.genome-engineering.org/crispr/), we generated the guide RNAs (sgRNAs) targeting the human miR-146a and BST-2/Tetherin gene were subcloned into the lentiCRISPRv2 vector. The gRNA sequence is 5’-TGGGTTGTGTCAGTGTCAGAC-3’ for miR-146a and 5’-GCTCCTGATCATCGTGATTC-3’ for BST-2/Tetherin. 6×10^6^ HEK-293T cells (human embryonic kidney cells) (ATCC) were transfected with 4*μ*g lentiCRISPRv2 containing gRNA, 3*μ*g psPAX2, and 2*μ*g pMD2.G to package virus. 1×10^5^ HT-29 cells were then infected with 100*μ*L lentivirus for two days. After limiting dilution, single clones were selected in the presence of puromycin (2*μ*g/mL) for three weeks and assayed for expression of the RNA by PCR or protein by Western blot.

### Exosome isolation, purification and labeling

Cell culture supernatants or patient serum samples were centrifuged at 10,000g for 45 mins to remove cell debris and the cleared samples were filtered through a 0.22μm filter. Exosomes were isolated using Exoquick-TC reagent or Exoquick (System Bioscience, USA), by following the manufacturer’s instruction. Briefly, the samples were gently mixed and incubated with Exoquick-TC reagent or Exoquick overnight at 4°C. Exosomes were precipitated by centrifugation at 1500 g at 4°C for 30 mins and re-suspended in phosphate buffered saline (PBS). To further purify exosomes, Exoquick-isolated exosomes were subjected to anti-CD63 antibody (Santa Cruz, USA) followed by corresponding secondary antibody coupled to magnetic beads (Miltenyi Biotec). The Miltenyi Biotec MidiMACS separator combined with LD columns (cat. #130-042-901) was used for exosome isolation. Exosomes isolated from HT-29 cells transfected with miR-146a mimics-cy3 were labeled with green fluorescent dyes using a commercial kit (Exo-Green label, #EXOG200A-1, System Biosciences) according to the manufacturer’s instructions. Knockout cells were incubated with 5μg/ml labeled exosomes at 37°C for 2h. For Dil labeling, the DiI (1,1’-Dioctadecyl-3,3,3’,3’-tetramethylindocarbocyanine perchlorate; Sigma-Aldrich) was added into the exosome-PBS to 1 μM and incubated for 20 min before spin washing, followed by an additional wash to remove the excess dye. Live or fixed Hela cells were incubated with DiI-labeled exosomes for 24h. The fluorescence was visualized with Olympus FluoView FV10i confocal microscope (Tokyo, Japan).

### Co-immunoprecipitation analysis of exosome samples

Exosomes isolated from cell culture supernatants were lysed in immunoprecipitation (IP) lysis buffer (0.5% NP40, 150 mM KCl, 1 mM NaF, 25 mM Tris, 2 mM EDTA, protease inhibitors and 0.5 mM dithiothreitol). 60 μg of total exosomal protein was incubated with AGO2 antibody (Abcam) and GW182 antibody (Santa Cruz) at 4°C overnight and then washed three times with PBST buffer. Non-specific rabbit IgG (Santa Cruz) was used as an IP control. A mixture of Protein A/G PLUS-Agarose beads (Millipore) was added and the incubation was continued for an additional 30 minutes. After three washes, the immunoprecipitated protein-RNA complex was either used for Western blot analysis or RNA purification.

### Transient transfection of miRNA and siRNA

Cells were grown to logarithmic phase in 100mm plates with antibiotic-free medium the day before transfection. The miRNA or siRNA transfection was performed with Lipofectamine3000 (Life Technologies, USA) on cells of 30%~50% confluence according to the manufacturer’s protocol. The final concentrations of miRNA mimic, mimic-cy3, si-Rab27a or their negative controls (RiBoBio, Guangzhou, China) were 100nM. The effect of transfection was examined by quantitative RT-PCR and Western blot.

### Northern blot

Reverse transcription was performed using PrimeScript RT Master Mix for RT-PCR (TaKaRa). RT products were PCR-amplified using DreamTaq Green PCR Master Mix (Thermo Scientific) and amplify the EV71 RNA (this sentence is confusing). PCR primer sequences were as follow: Forward: 5’-GGGGTACCAGTGATATCCTGCAGACGGG-3’; Reverse: 5’-GAAGATCTATAGCCCCAGACTGTTGTCC-3’. miR-146a primer was purchased from RiboBio (Guangzhou, China). The PCR products were resolved on 1% agarose/TAE gels containing ethidium bromide.

### Animal experiments

For exosome treatment, exosomes were injected into the tail veins of 4-week-old male NOD/SCID/IL2Rγ null mice (4 mice, 5μg exosome per mice), and the mice were sacrificed 24 h later for tissue sections. For *in vivo* fluorescent imaging, Exo-Red-labeled exosomes (Exo-Red exosome RNA fluorescent label kit, #EXOR100A-1, System Biosciences) isolated from HT-29 or THP-1 cells (50μg of total protein) were injected intravenously into five-week-old male nude mice (BALB/c nu/nu), and the red fluorescence of the whole body was acquired by IVIS spectrum (Caliper Life Sciences). The mice were sacrificed 24 h later, organs prepared and the red fluorescence was quantified in brain, liver, heart, kidney, spleen, lung, stomach, and intestine. Radiant efficiency was measured using Living Image 3.1 software (Caliper Life Sciences).

### Clinical samples

Serum samples from healthy and EV71-infected children were collected from April 2016 to June 2016 at Nanjing Children’s Hospital. Etiologic diagnosis of the patients was confirmed by detection of EV71 using duplex real-time reverse-transcriptive PCR.

### Fluorescence *in situ* hybridization and imaging

Infected cells were fixed for 10 min with fixation buffer (75% methanol, 25% glacial acetic acid) and subsequently washed with PBS and pre-hybridization buffer. Subsequently, the cells were probed overnight at 37°C in hybridization buffer containing 10% dextran sulfate, 2 mM vanadyl-ribonucleoside complex, 0.02% RNAse-free BSA, 2x SSC, and 10% formamide with the EV71 RNA probe (RiBoBio, Guangzhou, China). After hybridization, the cells were washed twice for 30 minutes at 30°C using a wash buffer (10% formamide and 2x SSC). Nuclear was visualized by staining with DAPI (Sigma) and the cells were examined under a fluorescence microscopy and images were analyzed using an Olympus FluoView FV10i confocal microscope (Tokyo, Japan).

### Statistical analysis

Data from at least three independent experiments were presented as mean ± SEM using SPSS 19.0 statistical software. The differences among treatment groups were analyzed by Student's *t* test or One-way Analysis of Variance (ANOVA) followed by Student Newman Keuls (S-N-K) test. Differences were considered statistically significant at a *P* value of <0.05.

## Supporting information

S1 Fig EV71 infection increased exosome secretion. (A) Schematic presentation of Exoquick+CD63 immuno-magnetic selection for exosome purification. (B) EM images of exosomes purified from EV71-infected HT-29 cells. (C) Histogram displaying the size distribution of the purified exosomes as analyzed by NTA. (D, E) Quantification of exosomes in THP-1 (D) and HT-29 (E) cells infected with EV71, CA16, heat-inactivated EV71 or CA16 for 24h. Data are shown as mean±SEM of three independent experiments.
(TIF)

S2 Fig Characterization of infectivity of exosomal EV71 and free virus *in vitro* and *in vivo*. (A) Western blot analysis with antibody to EV71 structural(VP1,VP0,VP2) or non-structural proteins(3AB,3A,3C,3D) of the exosomes isolated from virus—infected HT-29 cells. (B) Northern blot analysis showing that the exosome-associated EV71 RNA resisted RNase H degradation. (C) Exosomes isolated from HT-29 cells were labeled with DiI (red) and were added to Hela cells seeded on coverslips for 6h. Confocal images were acquired from live and paraformaldehyde fixed Hela cells. (D, E) Northern blot (D) and quantitative real-time PCR (E) analysis of EV71 RNA expression was performed on RD cells infected with Exo-EV71 RNA or free EV71 virus. Data are the mean ± SEM of three independent experiments. (F) Recipient L929 cells exhibited high uptake efficiency when being treated with Exo-EV71, as compared to the free virus treatment. Data points presented are averaged from twelve different fields. (G) Light microscopic images of L929 cells infected with Exo-EV71 or free EV71 virus. Scale bar = 50μm. (H) Real-time PCR analysis of EV71 viral RNA copy numbers in L929 cells infected with Exo-EV71 or free EV71 virus. The cells were treated with an equal copy number of viral RNA from Exo-EV71 RNA and free EV71 virus. (I): Virus titers were determined in various organ tissues 24h after injection by fluorescence *in situ* hybridization, bar = 500μm. All data are presented as the mean±SEM of three independent experiments. (*p<0.05, **p<0.01)
(TIF)

S3 Fig IFNs impaired exosome secretion induced by EV71 infection. (A) miR-146a genome knockout (GKO) HT-29 cell line (HT-29-146a^-/-^) was generated using the Clustered Regulatory Interspaced Short Palindromic Repeat (CRISPR)/CRISPR-associated protein 9 (CAS9) technology. Northern blot analysis showing that miR-146a expression was completely absent in HT-29-146a^-/-^ cells, in contrast to that in WT cells. (B) Real-time PCR analysis of the copy numbers of miR-146a in WT or HT-29-146a^-/-^ cells. (C, D) Quantification of exosomes isolated from WT and HT-29-146a^-/-^ cells, or HT-29-146a^-/-^ cells infected with EV71 at 0.05 TCID_50_, as measured by NTA (C) and Western blot (D). Data are shown as mean±SEM of three independent experiments.
(TIF)

S4 Fig EV71 infection induced preferential exosomal sorting of miR-146a. (A) Fold-change of selected miRNAs in HT-29 cells infected or mock-infected with EV71. (Fold change = 2^-ΔΔCt^ method, with Ct values normalized to U6; mean ± SEM, n = 3). (B) Effect of specific siRNA treatment on viral structural protein VP1 expression as determined by Western blot. HT-29 cells were transfected with individual miRNA-inhibitors at a final concentration of 100nM for 24h, followed by EV71 infection. VP1 was probed with a specific antibody. (C, D) Real-time PCR analysis showing the copy numbers of miR-146a per exosome isolated from Hela (C) and THP-1(D) cells infected or mock-infected with EV71. All the data are shown as mean±SEM of three independent experiments.
(TIF)

S5 Fig Exosomes derived from infected cells contained EV71 RNA in complex with AGO2-GW182-miRNA. (A) Immunoprecipitation of Ago2 and GW182 complex from exosome lysate isolated from culture supernatants of EV71 infected THP-1 cells at 0.1 TCID_50_. Normal non-specific rabbit IgG was used as a control antibody. (B and C) RNA ChIP analyses of Ago2-GW182 complexes in exosomes isolated from culture supernatants of EV71 infected THP-1 cells were subjected to Ago2 and GW182 pull down then total RNA isolation which was analyzed for miR-146a(B) or EV71 RNA(C) by PCR. Normal non-specific rabbit IgG was used as a control antibody.
(TIF)

S1 Table Differentially expressed miRNA in the exosomes from EV71-infected HT-29 cells by small RNA deep sequencing. (XLSX)

S2 Table Differentially expressed mRNA in the miR-146a^-/-^ HT-29 cells by real-time RT-PCR assay using human miR-146a targets RT^2^ Profiler PCR Array. (XLSX)
