# Supplementary figures and images for: Exosome-mediated miR-146a transfer suppresses type I interferon response and facilitates EV71 infection
Source: PLoS Pathog. 2017 Sep 14;13(9):e1006611. doi: 10.1371/journal.ppat.1006611 (PMC5614653; doi:10.1371/journal.ppat.1006611)

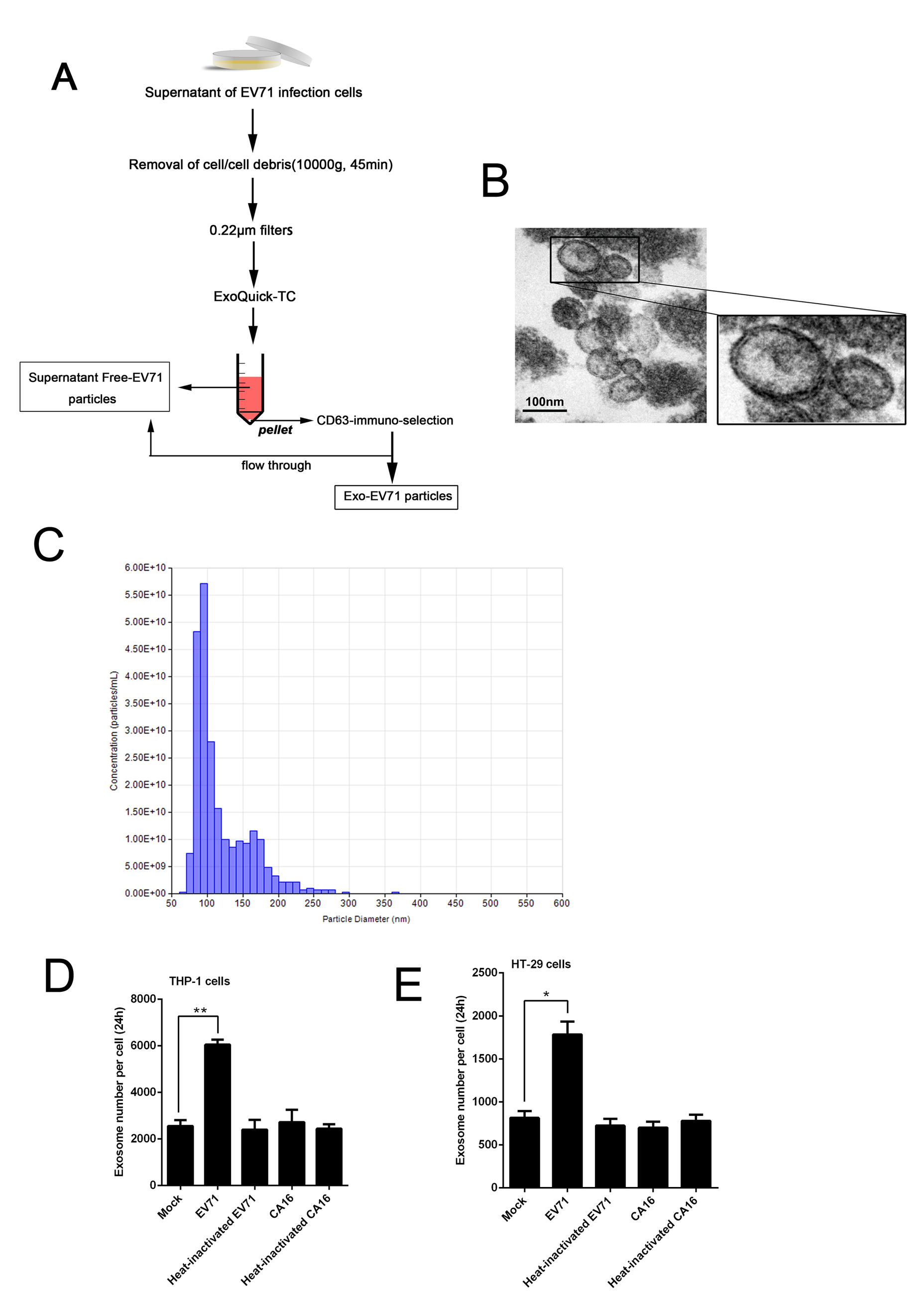

Supplement: S1 Fig — (A) Schematic presentation of Exoquick+CD63 immuno-magnetic selection for exosome purification. (B) EM images of exosomes purified from EV71-infected HT-29 cells. (C) Histogram displaying the size distribution of the purified exosomes as analyzed by NTA. (D, E) Quantification of exosomes in THP-1 (D) and HT-29 (E) cells infected with EV71, CA16, heat-inactivated EV71 or CA16 for 24h. Data are shown as mean±SEM of three independent experiments. (TIF) [file ppat.1006611.s001.tif]

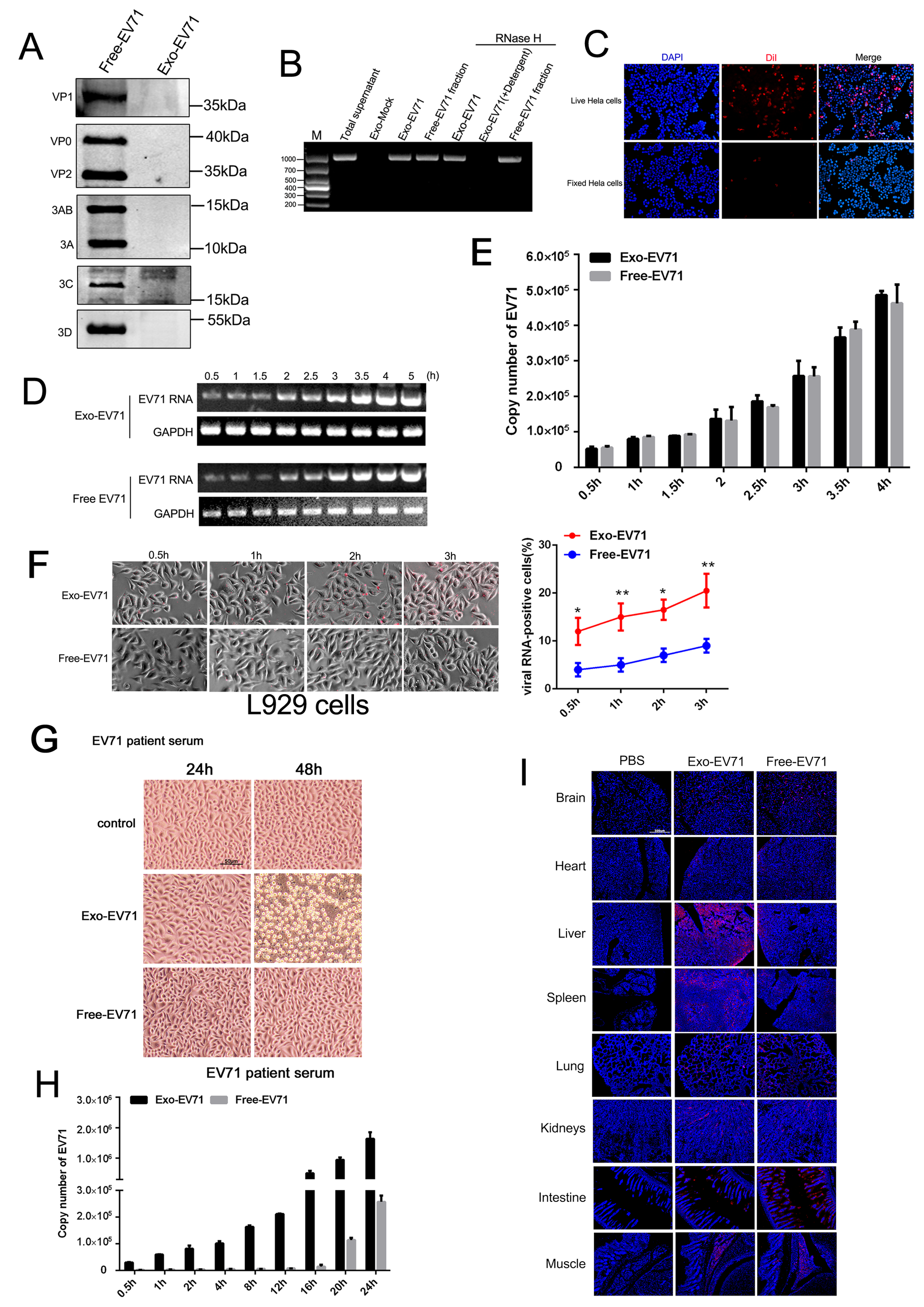

Supplement: S2 Fig — (A) Western blot analysis with antibody to EV71 structural(VP1,VP0,VP2) or non-structural proteins(3AB,3A,3C,3D) of the exosomes isolated from virus—infected HT-29 cells. (B) Northern blot analysis showing that the exosome-associated EV71 RNA resisted RNase H degradation. (C) Exosomes isolated from HT-29 cells were labeled with DiI (red) and were added to Hela cells seeded on coverslips for 6h. Confocal images were acquired from live and paraformaldehyde fixed Hela cells. (D, E) Northern blot (D) and quantitative real-time PCR (E) analysis of EV71 RNA expression was performed on RD cells infected with Exo-EV71 RNA or free EV71 virus. Data are the mean ± SEM of three independent experiments. (F) Recipient L929 cells exhibited high uptake efficiency when being treated with Exo-EV71, as compared to the free virus treatment. Data points presented are averaged from twelve different fields. (G) Light microscopic images of L929 cells infected with Exo-EV71 or free EV71 virus. Scale bar = 50μm. (H) Real-time PCR analysis of EV71 viral RNA copy numbers in L929 cells infected with Exo-EV71 or free EV71 virus. The cells were treated with an equal copy number of viral RNA from Exo-EV71 RNA and free EV71 virus. (I): Virus titers were determined in various organ tissues 24h after injection by fluorescence in situ hybridization, bar = 500μm. All data are presented as the mean±SEM of three independent experiments. (*p<0.05, **p<0.01) (TIF) [file ppat.1006611.s002.tif]

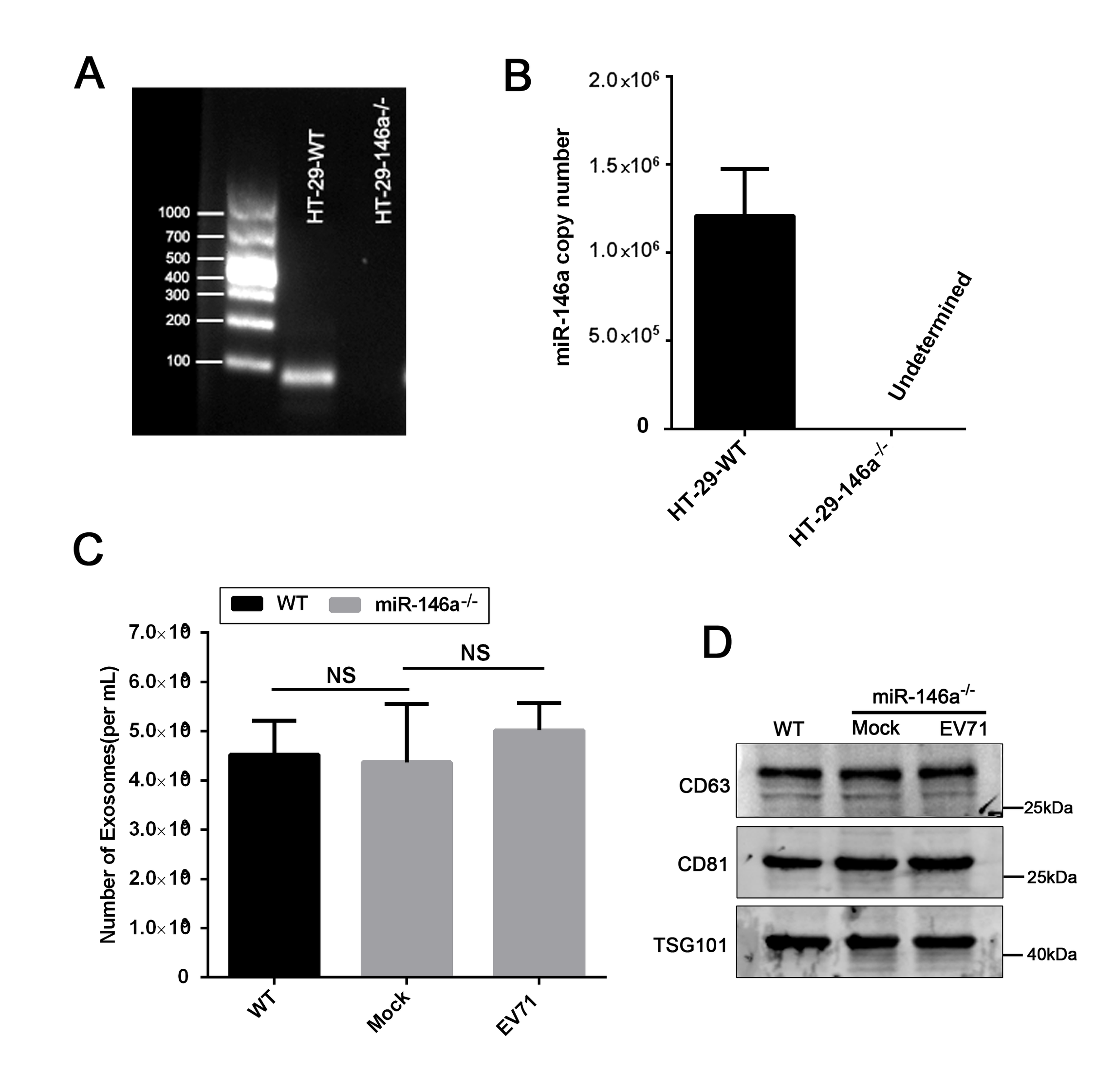

Supplement: S3 Fig — (A) miR-146a genome knockout (GKO) HT-29 cell line (HT-29-146a-/-) was generated using the Clustered Regulatory Interspaced Short Palindromic Repeat (CRISPR)/CRISPR-associated protein 9 (CAS9) technology. Northern blot analysis showing that miR-146a expression was completely absent in HT-29-146a-/- cells, in contrast to that in WT cells. (B) Real-time PCR analysis of the copy numbers of miR-146a in WT or HT-29-146a-/- cells. (C, D) Quantification of exosomes isolated from WT and HT-29-146a-/- cells, or HT-29-146a-/- cells infected with EV71 at 0.05 TCID50, as measured by NTA (C) and Western blot (D). Data are shown as mean±SEM of three independent experiments. (TIF) [file ppat.1006611.s003.tif]

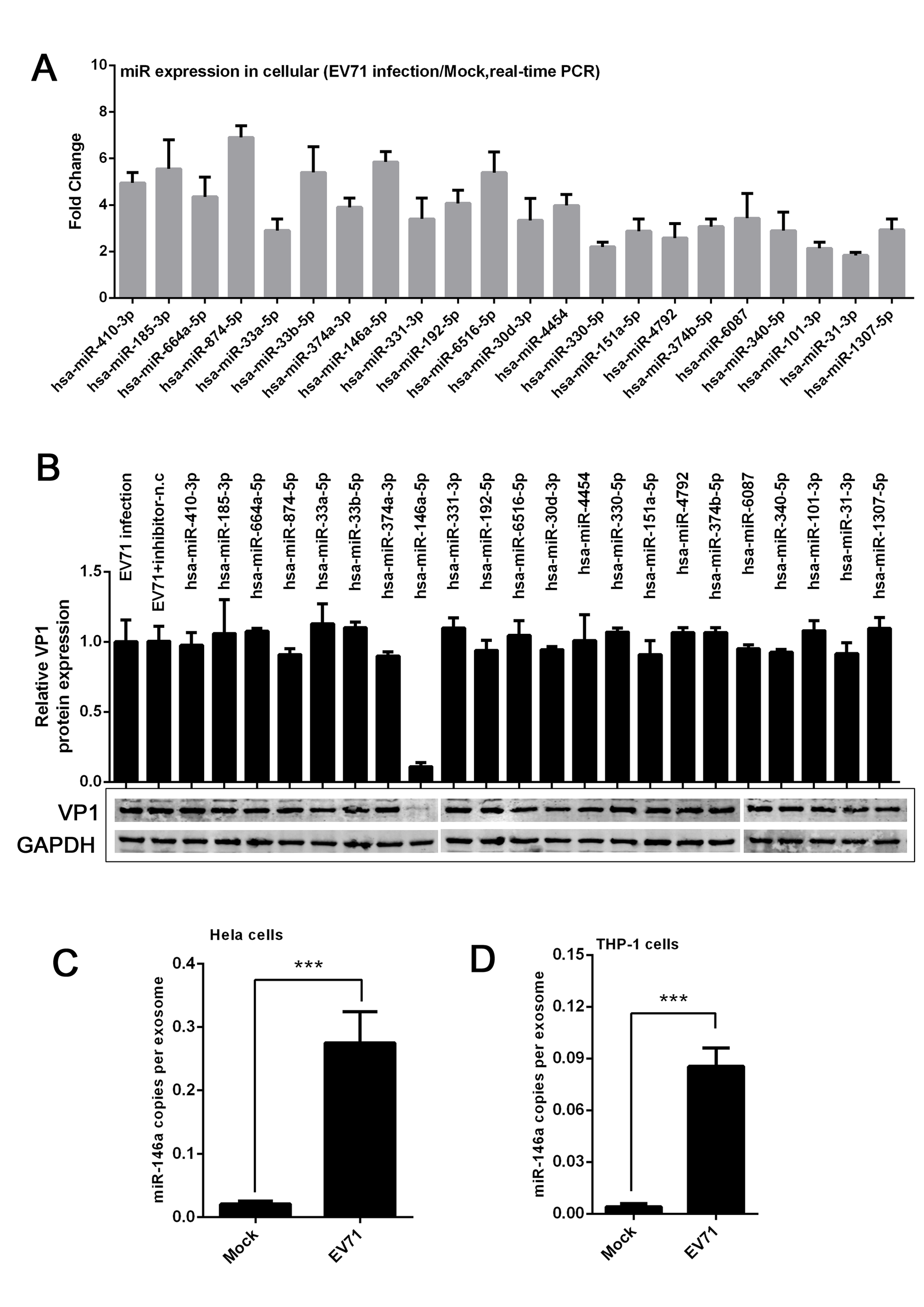

Supplement: S4 Fig — (A) Fold-change of selected miRNAs in HT-29 cells infected or mock-infected with EV71. (Fold change = 2-ΔΔCt method, with Ct values normalized to U6; mean ± SEM, n = 3). (B) Effect of specific siRNA treatment on viral structural protein VP1 expression as determined by Western blot. HT-29 cells were transfected with individual miRNA-inhibitors at a final concentration of 100nM for 24h, followed by EV71 infection. VP1 was probed with a specific antibody. (C, D) Real-time PCR analysis showing the copy numbers of miR-146a per exosome isolated from Hela (C) and THP-1(D) cells infected or mock-infected with EV71. All the data are shown as mean±SEM of three independent experiments. (TIF) [file ppat.1006611.s004.tif]

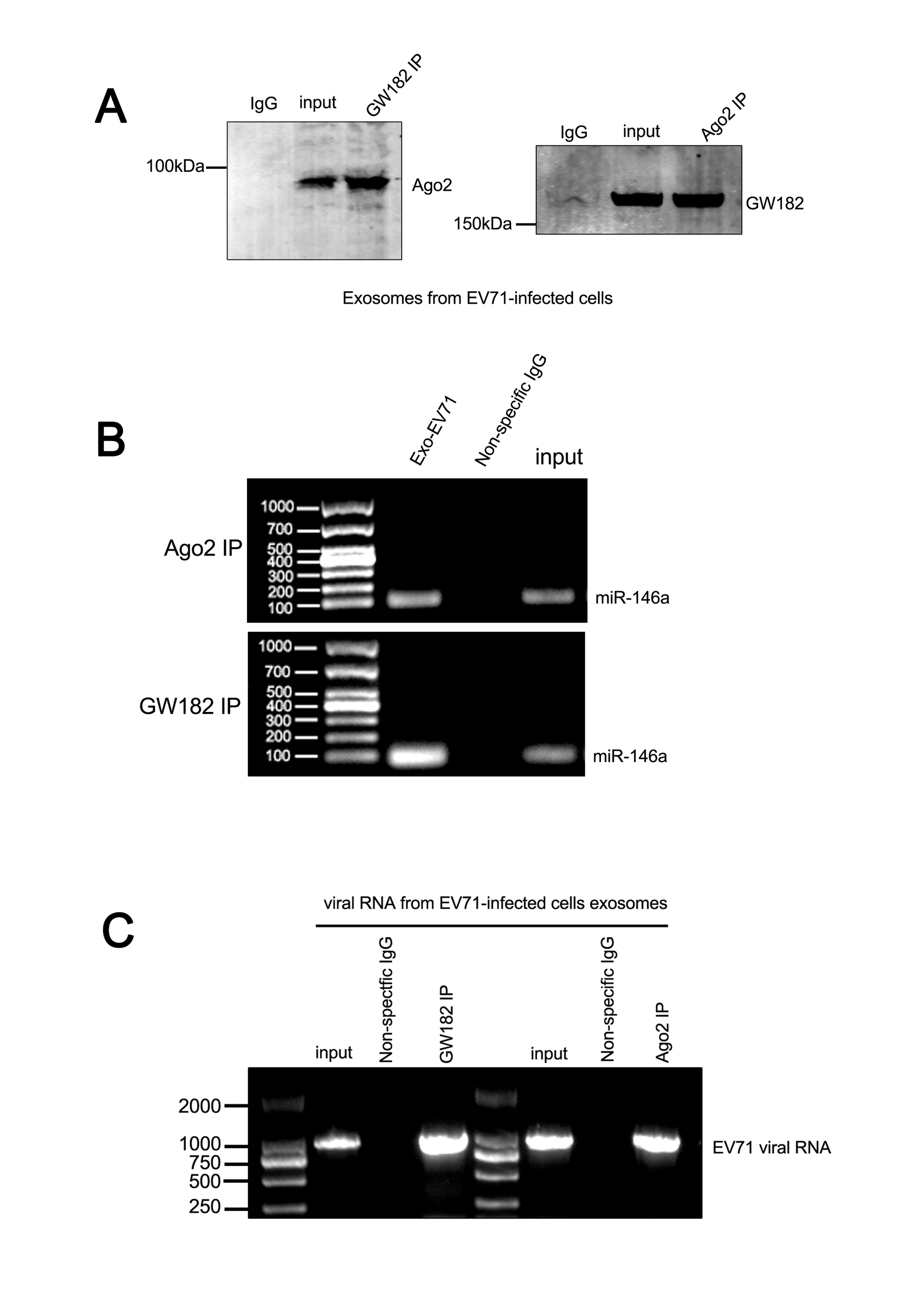

Supplement: S5 Fig — (A) Immunoprecipitation of Ago2 and GW182 complex from exosome lysate isolated from culture supernatants of EV71 infected THP-1 cells at 0.1 TCID50. Normal non-specific rabbit IgG was used as a control antibody. (B and C) RNA ChIP analyses of Ago2-GW182 complexes in exosomes isolated from culture supernatants of EV71 infected THP-1 cells were subjected to Ago2 and GW182 pull down then total RNA isolation which was analyzed for miR-146a(B) or EV71 RNA(C) by PCR. Normal non-specific rabbit IgG was used as a control antibody. (TIF) [file ppat.1006611.s005.tif]
